# Major-Effect Alleles at Relatively Few Loci Underlie Distinct Vernalization and Flowering Variation in Arabidopsis Accessions

**DOI:** 10.1371/journal.pone.0019949

**Published:** 2011-05-20

**Authors:** Amy Strange, Peijin Li, Clare Lister, Jillian Anderson, Norman Warthmann, Chikako Shindo, Judith Irwin, Magnus Nordborg, Caroline Dean

**Affiliations:** 1 Department of Cell and Developmental Biology, John Innes Centre, Norwich, England, United Kingdom; 2 Department of Molecular Biology, Max Planck Institute for Developmental Biology, Tübingen, Germany; 3 Department of Molecular and Computational Biology, University of Southern California, Los Angeles, California, United States of America; University of Oxford, United Kingdom

## Abstract

We have explored the genetic basis of variation in vernalization requirement and response in Arabidopsis accessions, selected on the basis of their phenotypic distinctiveness. Phenotyping of F2 populations in different environments, plus fine mapping, indicated possible causative genes. Our data support the identification of *FRI* and *FLC* as candidates for the major-effect QTL underlying variation in vernalization response, and identify a weak *FLC* allele, caused by a Mutator-like transposon, contributing to flowering time variation in two N. American accessions. They also reveal a number of additional QTL that contribute to flowering time variation after saturating vernalization. One of these was the result of expression variation at the *FT* locus. Overall, our data suggest that distinct phenotypic variation in the vernalization and flowering response of Arabidopsis accessions is accounted for by variation that has arisen independently at relatively few major-effect loci.

## Introduction

An important debate in evolutionary biology is the influence of few major-effect versus many minor-effect changes in the adaptation of organisms to different environments [1]. An important adaptive trait in plants is the timing of flowering. This significantly influences their fitness and so is tightly regulated, however, variation in this trait is required to enable plants to adapt to different environmental conditions. The regulatory network and molecular mechanisms mediating the impact of environmental cues on the timing of the floral transition have been extensively studied in Arabidopsis [2]. The data so far point to an integrated network of pathways that converge on a set of common targets to quantitatively regulate genes required to switch the vegetative apical meristem to a floral fate [2]. The natural variation in Arabidopsis flowering is extensive and several loci have been identified which contribute to this variation: *FRIGIDA (FRI)*, *FLOWERING LOCUS C* (*FLC*), *FLOWERING LOCUS M* (*FLM*), *CRYPTOCHROME 2*, *HUA2*, *PHYTOCHROME C* and *FLOWERING LOCUS T* (*FT*) [3], [4], [5], [6], [7], [8], [9], [10], [11], [12], [13], [14], [15]. We have focused on vernalization, the acceleration of flowering by a prolonged period of cold, namely winter. Different Arabidopsis accessions show variation in the length of cold required to satisfy the vernalization requirement and this correlates with the ability to epigenetically silence *FLC* [6]. Initial analysis of four F2 populations mapped the QTL contributing to the variation in *FLC* epigenetic silencing to broad genomic regions and concluded that, unexpectedly, none of them corresponded to the *trans-*factors currently known to regulate vernalization [10]. Further analysis was therefore required to identify the genes involved.

We have continued to explore the basis of variation in vernalization requirement and response in these four accessions, plus two additional accessions, with vernalization requirements but low *FLC* levels, from N. America. Our logic was that analysis of phenotypically distinct accessions might reveal independent adaptations of the vernalization process. We conclude that major-effect alleles at relatively few loci can provide the basis for adaptively important variation in Arabidopsis accessions.

## Results

### QTL profile in accessions selected for their distinct vernalization response

**Figure 1:**
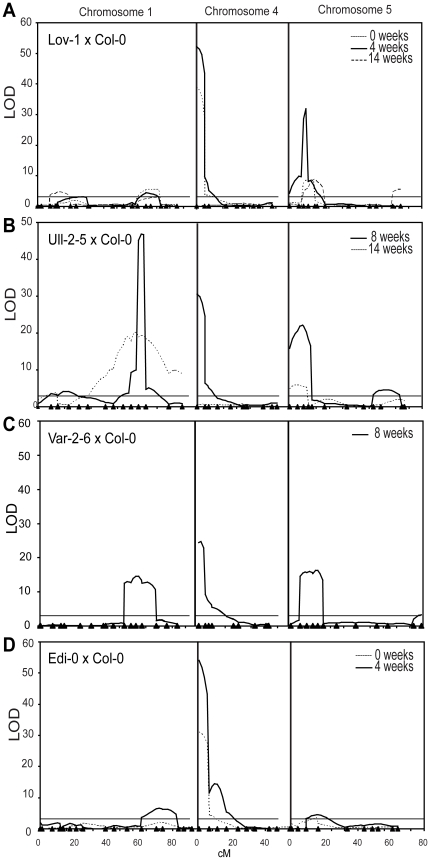
QTL analysis for variation in vernalization response. Composite interval mapping was used to identify genes contributing to the variation in vernalization response after treatment of Arabidopsis populations with different lengths of cold. (A) Lov-1 × Col-0, (B) Ull-2-5 × Col-0, (C) Var-2-6 × Col-0 and (D) Edi-0 × Col-0. Each chromosome with significant QTL (chromosome 1, 4 and 5) is shown separately and the positions (in cM) of the markers used are indicated as triangles. LOD (Logarithm of odds) scores were calculated by QTL Cartographer with a 5 % significance threshold (shown as dashed lines) determined from a 1000 permutation test. For (B) Ull-2-5 x Col this resulted in a high threshold due to segregation distortion, which is widespread in this cross (Figure S2). Each chromosome was tested individually and chromosome 3 identified as the cause of the high threshold. The permutation analysis was then performed excluding chromosome 3.

Four Arabidopsis accessions Lov-1, Ull-2-5, Var-2-6 and Edi-0 had previously been selected for QTL analysis [10]. Lov-1 was collected in N. Sweden from a rocky, south-facing slope on the Baltic coast (Lat/Long 62.5/18.1); Ull-2-5, from S. Sweden, was collected from a dry, sandy meadow that had not been tilled for 80 years (Lat/Long 55.3/14.2); Var-2-6, also from S. Sweden, was collected from a gravel beach in a nature reserve on the Baltic coast (Lat/Long 56.1/13.5) and Edi-0 collected from the Botanical Gardens in Edinburgh, Scotland (Lat/Long 56.0/3.0) [10]. The accessions had been selected as they showed particular features of interest in their vernalization response: Lov-1 is insensitive to 4 weeks of cold but responded strongly to five or more weeks of cold; Ull-2-5 is very late flowering even after extensive vernalization (10 weeks of cold); Var-2-6 is typical of many Scandinavian accessions showing a quantitative acceleration with increasing weeks of cold, saturating at 10 weeks; Edi-0 is very late-flowering when not exposed to low temperature but responded strongly to 4 weeks of cold. To record flowering time we counted total leaf number at flowering after specific treatments: Lov-1 × Col-0 and Edi-0 × Col-0 F2 seedlings were vernalized for 4 weeks, Ull-2-5 × Col-0 and Var-2-6 × Col-0 F2 seedlings were vernalized for 8 weeks. To obtain further phenotypic data from these populations the mean flowering time, based on days-to-flowering of F3 plants, was determined after no vernalization and saturating vernalization (14 weeks) (Fig. S1A–D). The QTL were mapped onto the genetic maps using Composite Interval Mapping (CIM) (Fig.1, Fig. S2, Table S1). Table 1 indicates the QTL position, strength and dominance, found in each population under the different conditions, plus potential candidate genes. The major QTL on chromosome 4 corresponds to the *FRI* gene and was expected given that Col-0, which has a non-functional *FRI*, was used as the recurrent parent [3]. *FRI* accounts for the highest percentage of the phenotypic variation in the Lov-1 × Col-0, Var-2-6 × Col-0 and Edi-0 × Col-0 populations. The additive allelic effect of, and variance explained, by *FRI* decreases with increasing vernalization (Fig. S3). This is most evident in the Lov-1 × Col-0 population; with no vernalization it explains 68 % of the variance, after a 4 week vernalization this is reduced to 48 %, and with a 14 week vernalization it is no longer significant. Interestingly, in the Edi-0 × Col-0 population, there is a second QTL at 13.9 cM on chromosome 4 (Fig. 1D), which might account for the rapid vernalization response of Edi-0, however, this region contains no obvious candidate flowering time genes. A QTL in a similar position has been identified in a RIL population derived from a cross between accessions Nok-3 and Ga-0 [14].

**Table 1:** QTL characteristics and candidate genes mapping to the interval.

	Accession × Col	Weeks V	QTL Peak cM	LOD	A	D	R^2^x100	Candidate genes
Chr. 4	Lov-1	0	0.01	39.06	54.96	10.85	68.19	*FRI*
		**4**	**0.01**	**52.24**	**37.99**	**22.08**	**48.39**	*FRI*
		14	0.01	ns				
	Ull-2-5	**8**	**0.01**	**30.52**	**12.49**	**6.86**	**20.8**	*FRI*
		14	0.01	ns				
	Var-2-6	**8**	**0.01**	**24.70**	**7.64**	**7.05**	**34.92**	*FRI*
	Edi-0	0	0.01	31.60	23.16	7.93	73.88	*FRI*
		**4**	**0.01**	**54.01**	**15.56**	**11.62**	**66. 9**	*FRI*
		**4**	**13.9**	**30**	**nd**	**nd**	**nd**	
	Kno-18	**0**	**0.01**	**28.51**	**48.44**	**52.76**	**43**	*FRI*
	RRS-10	**8**	**0.01**	**11.15**	**8.86**	**4.88**	**14.46**	*FRI*
Chr. 5	Lov-1	0	16.21	8.85	27.89	5.41	13.83	*FLC, FY, AGL15, CO, COL1*
		**4**	**12.6**	**31.93**	**37.16**	**12.44**	**33.13**	*FLC*
		14	19.21	8.87	1.81	−0.39	25.23	*FLC, FY, AGL15, CO, COL1, FRL1, LHP1*
	Lov-1	14	82.11	5.57	−1.42	−0.29	16.92	*VIN3, VIP4, TOC1, ELF5, LFY, MAF2*-*5*
	Ull-2-5	**8**	**9.81**	**22.19**	**12.0**	**−2.67**	**18.1**	*TFL1, ELF6, FLC, FY, AGL15, CO, COL1, FRL1, LHP1*
		**8**	**72.70**	**4.56**	**5.65**	**−1.61**	**4.2**	*FRL3,EMF2*
		14	5.81	5.92	1.52	−0.63	10.46	*TFL1, ELF6, FLC, FY*
	Var-2-6	**8**	**18.51**	**16.34**	**6.18**	**−0.62**	**18.57**	*FLC, FY, AGL15, CO, COL1*
	Edi-0	0	18.91	3.53	6.60	1.01	2.71	*HUA2*
		**4**	**18.37**	**4.52**	**2.34**	**2.12**	**3.59**	*FLC, FY, AGL15, CO, COL1, FRL1, LHP1, HUA2*
	Kno-18	**0**	**14.4**	**7.51**	**−38.7**	**3.46**	**15.92**	*FLC, FY, AGL15, CO, COL1, FRL1, LHP1, HUA2*
	RRS-10	**8**	**10.8**	**24.5**	**14.39**	**−5.9**	**39.26**	*FLC, FY, AGL15, CO, COL1, FRL1, LHP1, HUA2*
Chr. 1	Lov-1	14	15.21	4.66	0.92	−0.47	13.49	*SEX1*
	Lov-1	0	89.41	5.60	16.80	2.37	8.85	*VIP5, LDL1, FT, FKF1*
		**4**	**81.9**	**4.44**	**17.00**	**0.82**	**4.15**	*VIP5, LDL1, FT, FKF1*
		14		ns				
	Ull2-5	**8**	**77.81**	**46.93**	**11.94**	**−5.06**	**43.17**	*LDL1, FT*
		**8**	**21.11**	**4.16**	**3.38**	**−0.33**	**1.9**	*GI, SEP3*
		14	74.21	20.18	2.05	−0.40	24.44	*SPL4, VIP5, LDL1, FT, FKF1*
	Ull-2-5	14	13.91	3.91	1.25	−0.87	9.08	*SEX1*
	Var-2-6	**8**	**74.61**	**14.58**	**7.33**	**0.48**	**20.82**	*SPL4, VIP5, LDL1, FT, FKF1*
	Edi-0	0		ns				
		**4**	**82.81**	**6.67**	**7.57**	**−5.33**	**9.86**	*VIP5, LDL1, FT, FKF1*
	Kno-18	**0**	**77.5**	**5.04**	**28.23**	**−17.2**	**12.2**	*VIP5, LDL1, FT, FKF1*
	RRS-10	**8**	**40.9**	**2.54**	**3.79**	**2.15**	**4.53**	*FRL2*

A: Additive effect of the QTL i.e. the contribution of one accession allele to the phenotypic variation. Values are shown with respect to the non-Col allele.

D: Dominance effect of the QTL i.e. the deviation of the heterozygote phenotype from that expected based on the additive effect. Values are shown with respect to the non-Col allele.

R^2^: Phenotypic variation explained by the QTL.

ns not significant.

Bold numbers are flowering time based on leaf number, rest are flowering time based on days to flowering measured when the inflorescence reached 3 cm

Shoulder on chromosome 4 QTL in Edi-0 population is associated with a high number of apparent double recombinants in the region – no clear candidate maps to this interval.

The extent of the flowering variation attributed to the *FRI* QTL varied between populations and the flowering time of individuals homozygous for the four active *FRI* alleles differed (Fig. S3A). This might correlate with the slight amino acid variation in FRI; Edi-0 and Var-2-6 have common FRI alleles differing in two amino acids compared to Lov-1, G146E and M148I, while Ull-2-5 has R74C and D167E, compared to Lov-1. It will be interesting to test whether these amino acid differences affect FRI function [16]. This could also be explained by differing expression levels of *FRI* alleles amongst the accessions; Var-2-6 *FRI* being expressed more strongly than the other *FRI* alleles. [6].

### Multiple QTL are resolved on chromosome 5

The different vernalization treatments resolved multiple QTL on chromosome 5. The major QTL, detected in populations that had not been vernalized or had a short period of vernalization, covered the region containing *FLC* (At5g10140). *FLC* is a likely candidate gene as our previous analysis had shown that the stability of *FLC* epigenetic silencing differed in the accessions, with some requiring a much longer period of cold than others before stable silencing was achieved [10]. The effect of this QTL was maximal in populations that had experienced a short vernalization period (Table 1). However, the breadth of the QTL in the Ull-2-5 × Col-0 population and the complex profile of the QTL in the Lov-1 × Col-0 population treated with 4 weeks of cold suggested additional genes contribute to the variation. In Lov-1 × Col-0, the QTL peak was centred over *FLC* in plants given a 4 week vernalization but the QTL peak mapped several cM away after a 14 week vernalization (Fig. 1A). F2 individuals were backcrossed four times to Col-0 carrying an active *FRI* allele to dissect the QTL. Analysis of recombinants suggested at least two closely linked QTL in this region, one was mapped in the *FLC* interval ∼1.5–3.4 Mb – where *FLC* is at 3.17 Mb, with a second one mapped between 4 and 6 Mb (Fig. S4B, Table S2). This second interval contains several possible candidates: *FY* [17], *AGL15* [18], *FRL1* [19], *LHP1*/*TFL2* [20], *CONSTANS* (*CO*) /*CONSTANS*-*LIKE 1* (*COL1*) [21] (Table 1).

The QTL positioned on chromosome 5 in the Edi-0 × Col-0 population maps near to *HUA2* (Fig. 1D). The Edi-0 allele in this chromosomal region causes lateness, therefore if it corresponds to *HUA2* it is likely to be a gain-of-function allele similar to that found previously in accession Sy-0 [12]. Potential candidate genes for other QTL on the lower arm of chromosome 5 include *VIN3, VIP4, TOC1, ELF5*, *LEAFY* and the *MADS AFFECTING FLOWERING* gene family *(MAF2-5)* (Table 1); similar QTL have been found in other studies [14], [22], [23], [24], [25], [26].

### The major QTL on chromosome 1 is caused by *FT* expression variation in Ull-2-5

**Figure 2:**
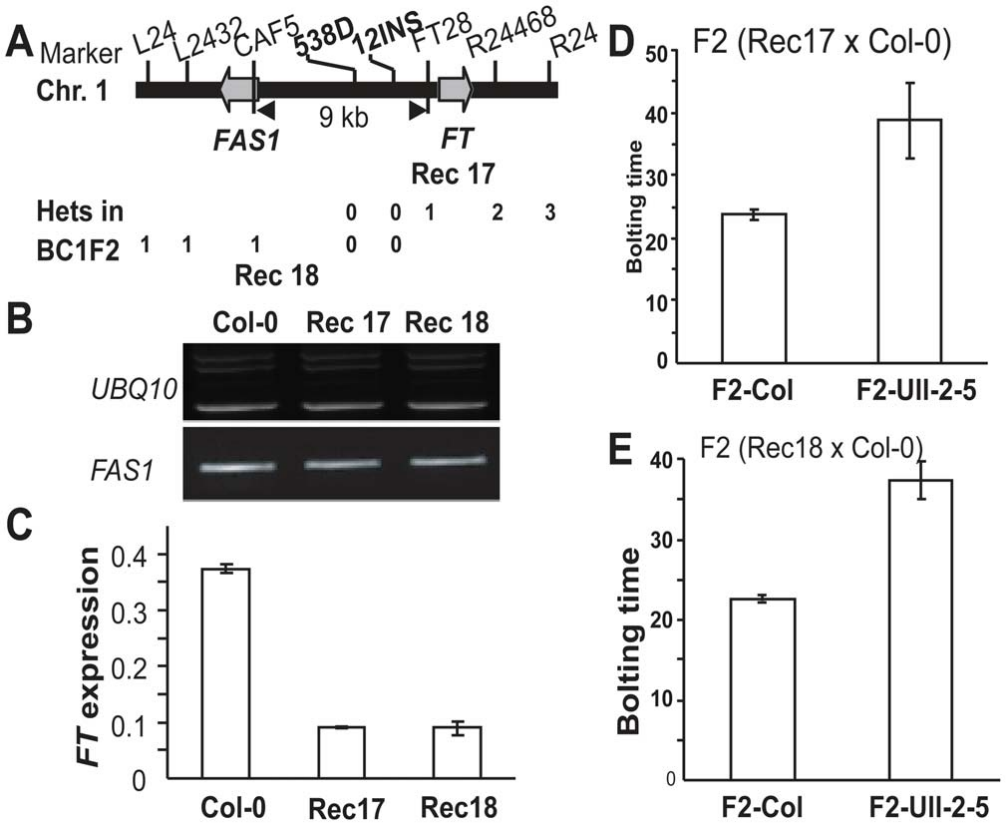
Fine-mapping and allelic analysis of the QTL on chromosome 1. (A) Fine-mapping of the QTL – the later flowering time variation co-segregated with marker 538D and 12INS. (B) *FAS1* expression in Col-0 and two recombinants plants Rec17 and Rec18. (C) *FT* expression in Col-0, Rec17 and Rec18. *FT* expression level was normalized to *UBC*. (D and E) Segregation analysis of the F2 population obtained from Rec17 (D) and Rec18 (E) crossed to Col-0 respectively. Error bars in (B) and (C) show S. D. from three experimental replicates, in (D) and (E) shows S. D. of at least 20 individuals.

A QTL at ∼24 Mb on chromosome 1 appears common to most of the populations (Fig. 1). This was differentially affected in the various populations by the length of vernalization and is no longer significant after a 14 week vernalization in the Lov-1 × Col-0 population. In contrast, it is the principal QTL in the Ull-2-5 × Col-0 population, accounting for 43 % and 24 % of the variance after an 8 week and 14 week vernalization respectively (Table 1). In the Edi-0 × Col-0 population it is only significant after a 4 week vernalization. The candidate gene for this QTL is *FT* (At1g65480), or linked genes (for example, *LDL1, SPL4, VIP5, FKF1*). *FT* has been the focus of many recent studies as FT protein appears to function as the physiologically described ‘florigen’ [27] moving from the leaf phloem to the apical meristem to promote floral transition [28], [29], [30]. To identify the causative gene, we developed a mapping population from the Ull-2-5 × Col-0 F3 lines. We selected lines that were late flowering and carried the Ull-2-5 allele in the major QTL region on chromosome 1, and Col-0 alleles in the QTL regions on chromosome 4 and chromosome 5, and backcrossed them to Col-0. The QTL was mapped to a 9 kb interval between markers CAF5 and FT28, which included the upstream region of *FT* and a small part of the linked *FAS1* gene (At1g65470) (Fig. 2A, Table S2). The entire genomic region was sequenced (deposit number: GQ370818) and compared to the Col-0 sequence. Three nucleotide changes were found in the Ull-2-5 *FAS1* gene, located in introns, whilst two synonymous polymorphisms and seven intronic polymorphisms were found in *FT*. Multiple single nucleotide differences and several large indels were found in the intergenic region between *FAS1* and *FT* (Table S3). Analysis of *FAS1* and *FT* expression in different recombinants showed that the 9 kb Ull-2-5 genomic region common in two recombinant plants, Rec17 and Rec18 (Fig. 2B, C) contained the causative polymorphism. Rec17 and Rec18 were crossed to Col-0 to generate F2 populations. In each population, the progeny were genotyped into F2-Ull-2-5 and F2-Col groups using marker 12INS. The Ull-2-5 allele was clearly associated with late flowering and the Col-0 allele with early flowering, confirming that the regulatory region of *FT* is underlying the QTL on chromosome 1 (Fig. 2D, E). These results indicated that the natural allelic variation of Ull-2-5 is through regulation of *FT* expression and not in protein function. Recently, Schwartz and colleagues also mapped a flowering time QTL in the *FT* regulatory region in a population from a cross between Est-1 x Col-0 [15]. We therefore compared the *FT cis*-regulatory sequences between the Ull-2-5 and Est-1 alleles and although these two alleles are very distinct, with only short regions in common, and different to those in Col-0 (Table S3), they both influence the *FT* expression profile.

**Figure 3:**
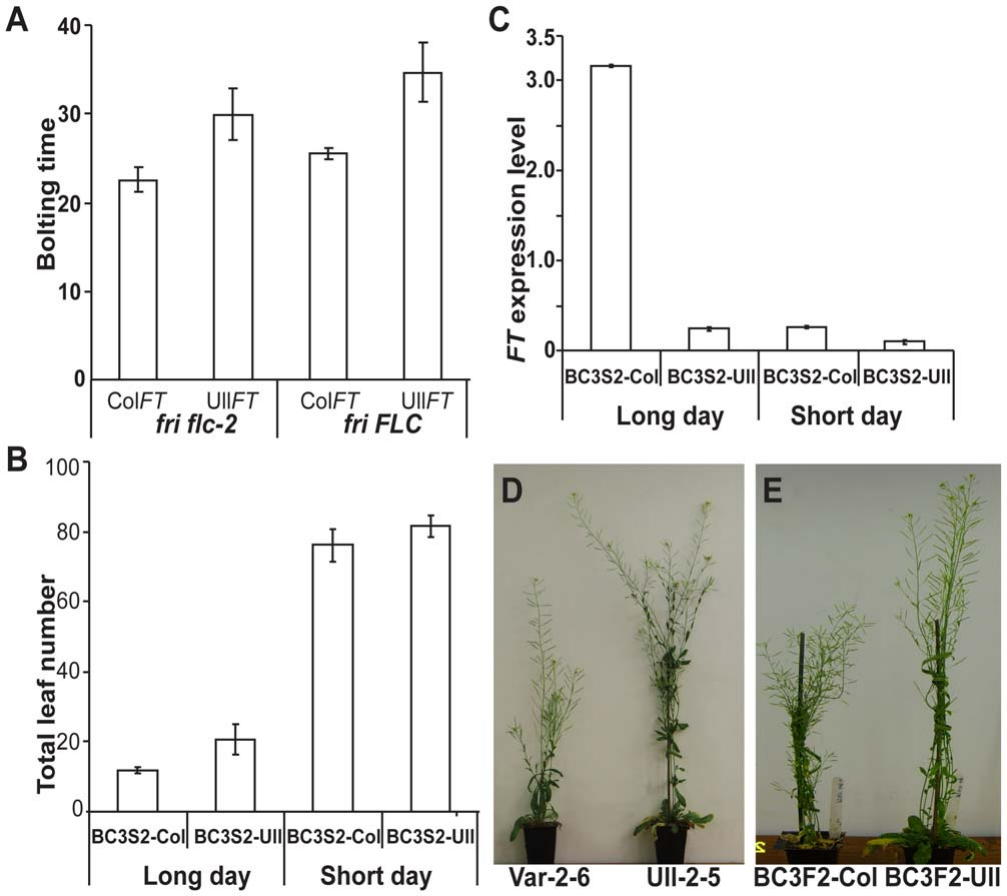
Functional analysis of the Ull-2-5 *FT* allele using backcrossed populations. (A) Comparison of the contribution of Ull-2-5 and Col-0 *FT* alleles to flowering time with or without a functional FLC. (B) Comparison of flowering time between BC3S2-Ull and BC3S2-Col in long and short day growth conditions. (C) *FT* expression of Col-0 and Ull-2-5 alleles in response to different day lengths (D) Final size of plants vernalized for 10 weeks and then grown in a greenhouse. (E) Plant size of BC3S2-Col (left) and BC3S2-Ull (right) in long day growth condition. Error bars in (A) show S. D. of 20 individual plants, in (B) and (C) they show S. D. from three experimental replicates.

Since variation in *FLC* silencing is also associated with these accessions we asked whether the delayed flowering caused by the *FT* regulatory polymorphism was dependent on *FLC* down-regulation. Backcrossed Ull-2-5 plants were crossed to the *flc-2* mutant [31], and the flowering time of F2 plants with different genotypes was determined. The Ull-2-5 *FT* allele delayed flowering irrespective of whether a functional *FLC* or non-functional *flc-2* allele was present (Fig. 3A), suggesting this *FT cis*-element variation does not require *FLC*–mediated *FT* regulation. We therefore asked whether this variation affected *FT* response to photoperiod. Groups of plants homozygous for either Ull-2-5 *FT* or Col *FT* (from a self of the third backcross BC3S2-Ull and BC3S2-Col selected using marker 12INS) were selected. BC3S2-Ull plants flowered much later than BC3S2-Col under long day (16 h) growth conditions, but in short days (8 h) the difference in flowering time was subtle (Fig. 3B). This is similar to the behaviour of the *ft* mutant whose late flowering phenotype largely disappears in short day conditions [32]. In addition we found *FT* expression level in BC3S2-Ull was much lower than that of BC3S2-Col in long day conditions, but in short days the *FT* expression level in both genotypes was quite low (Fig. 3C). This reduced induction of the Ull-2-5 *FT* allele by long day photoperiods resulted in later flowering of Ull-2-5. In general, late-flowering Arabidopsis plants remain in the vegetative phase longer thus producing more leaves and larger inflorescences. Consistent with this, mature, flowering Ull-2-5 plants were found to be much larger and more robust than the other accessions in this study (Fig. 3D), and in the population of selfed BC3 plants, BC3S2-Ull individuals were also more robust than BC3S2-Col (Fig. 3E). Ull-2-5 was collected from a population growing in a meadow that has been undisturbed for approximately 80 years (Nordborg M, unpublished). It is possible that this robust, late-flowering character, caused by variation at *FT*, may facilitate competition with other plants and so be beneficial for the fitness of Arabidopsis in this particular habitat.

Minor QTL on chromosome 1 are present in the Lov-1 and Ull-2-5 populations after 8 and 14 weeks vernalization. Whether these represent variation in the same or different genes was not established but several flowering time genes map to this region, including *SEX1*, which we have found influences flowering time (CL and CD unpublished) (Fig. 1A). The QTL on the lower arm became much broader when given 14 weeks' vernalization compared to that of 8 week's vernalization, covering more candidate genes around *FT*, including *SPL4*, *VIP5*, *LDL1* and *FKF1*.

### Two accessions from N. America with novel variation in vernalization requirement and response

Two accessions from N. America, Kno-18 and RRS-10 flower relatively late but have low *FLC* expression despite putatively functional *FRI* alleles [6]. RRS-10 also responds relatively poorly to 8 weeks of vernalization [6]. To investigate if additional floral repressors might be functioning in these accessions, Kno-18 and RRS-10 were analysed. RRS-10 and Kno-18 are closely related, originating probably from a recent European founder event [33], [34], [35]. However, they are two of the least closely related N. American accessions from the Nordborg 96 set [36]. F2 populations were generated from the two accessions after crossing to Col-0 and flowering time assayed by total leaf number (Fig. S1E, F). The Kno-18 × Col-0 F2 population was grown with no vernalization to identify the loci accounting for the discrepancy between low *FLC* expression and the late flowering phenotype. The RRS-10 × Col-0 F2 population was given 8 weeks of vernalization to analyse the basis of reduced vernalization response.

**Figure 4:**
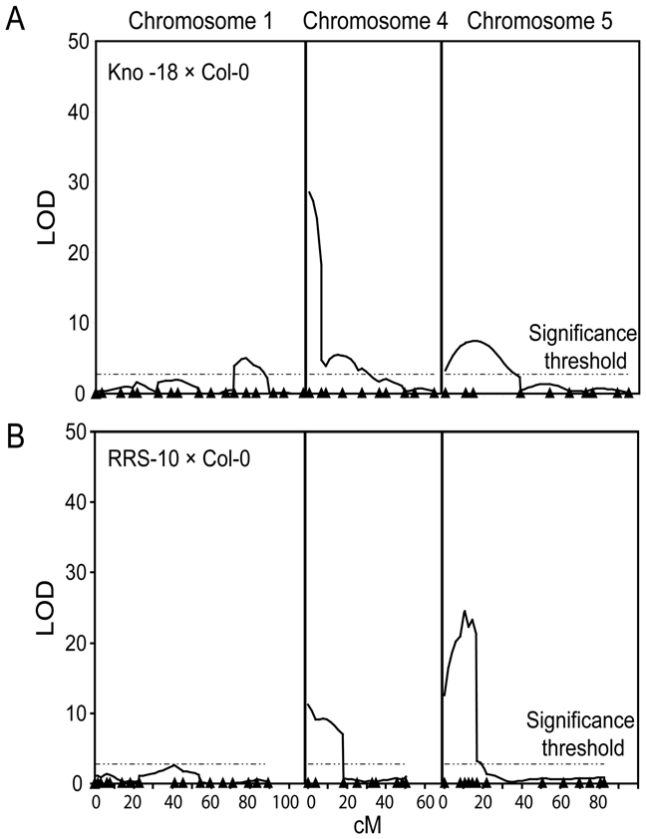
QTL analysis of vernalization requirement and response in two accessions from N. America. QTL were found on chromosome 1, 4, and 5 (chromosomes 2 and 3 not shown). Dashed line shows 5 % significance threshold, as calculated from a 1000 permutation test. The positions (in cM) of the markers used are indicated as triangles. (A) KNO-18 × Col F2 population scored for flowering time without vernalization. (B) RRS-10 × Col F2 population scored for flowering time after 8 weeks of vernalization.

The expected *FRI* QTL appeared on chromosome 4 in both populations accounting for 43% of the variance in the Kno-18 × Col-0 population and approximately 15% in the RRS-10 × Col-0 population, despite 8 weeks of vernalization (Fig. 4, Table 1). A shoulder to the *FRI* QTL, in a similar position to that in Edi-0 (∼13 cM) was also found in both populations and may represent the contribution of an unknown gene on chromosome 4. Possible minor effect QTL were found on chromosome 1 in both populations. The Col-0 alleles in both cases conferred earliness, which was recessive in the Kno-18 × Col-0 population and semi-dominant in the RRS-10 × Col-0 population (Figs. S5B, S6B). In the Kno-18 × Col-0 population the QTL mapped near *FT*, perhaps consistent with a less-responsive *FT* allele in the Kno-18 accession contributing to late-flowering (Fig. 4A, Fig. S5A). In the RRS-10 × Col-0 population the QTL mapped near *FRL2*, which is an interesting candidate given the relatively poor vernalization response of RRS-10 (Fig. 4B) [37].

A QTL on chromosome 5 mapped close to *FLC* in both populations but the alleles conferred different flowering time phenotypes, depending on the cross and environmental condition. In non-vernalized individuals the Kno-18 *FLC* allele conferred earlier flowering than the Col-0 allele (Fig. S5C). In contrast, in individuals vernalized for 8 weeks, the RRS-10 *FLC* allele (and linked genes) conferred later flowering than those carrying the corresponding Col-0 alleles (Fig. S6C).

**Figure 5:**
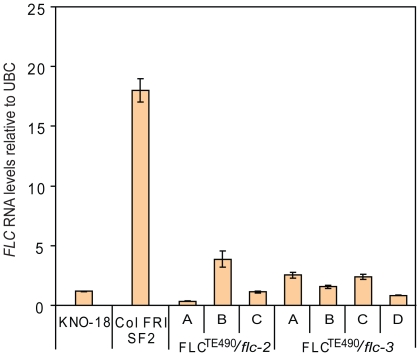
*FLC* expression level of Col *FRI flc* lines containing *FLC^TE490^* compared to controls. *FLC* RNA levels measured by qRT-PCR and normalised to *UBC*. Error bars show standard error from three experimental replicates.

To further analyse allelic diversity at *FLC* and continue investigating the basis of the QTL, the *FLC* alleles, including 2,769 bp upstream of the *FLC* start codon and 1,344 bp downstream of the stop codon, were sequenced from both accessions. Kno-18 and RRS-10 were found to have identical *FLC* alleles (Fig. S7). The SNPs between the Col-0 and Kno-18/RRS-10 alleles are distributed throughout the regions of *FLC* which have been previously identified as important for regulation; one SNP in particular is in a putative b-ZIP binding domain in the *FLC* promoter [38]. In addition to the numerous SNPs, the Kno-18 and RRS-10 *FLC* alleles contain a 1.19 kb insertion at +490 bp in intron 1 (*FLC^TE490^*). This insertion has 95.5 % similarity to the MULE (Mutator-like element) transposable element (TE) found in the Landsberg *erecta* (L*er*) *FLC* allele [39], however the TE in RRS-10 and Kno-18 is in the opposite orientation to that in the L*er FLC*, and has a different insertion site. It is flanked by a 9 bp sequence 5′- TTTCATTAT -3′ resembling a target site duplication, which is only present once in Col-0 *FLC* (Fig. S8). Five other accessions in the Nordborg 96 set, all from N. America (Yo-0, PNA-10, RMX-A02, Kno-10 and Dem-4) have the same 1.19 kb insertion as RRS-10 and Kno-18. To check if the transposon insertion causes the changed *FLC* expression, the TE was cloned from Kno-18 and inserted into Col-0 *FLC* to create a chimeric *FLC* allele, known as *Col FLC^TE490^*, and introduced by transformation into Col *FRI flc*-2. The *FLC* expression of the *Col FLC^TE490^* transformed lines was comparable to Kno-18 (Fig. 5). Corresponding to low *FLC* levels in non-vernalized plants the transformants all flowered early, averaging 15 leaves and this was not significantly affected by vernalization. Therefore the late-flowering of RRS-10 and Kno-18 would appear to be the result of a small contribution (4–12 %) from the chromosome 1 QTL and additional genes under the chromosome 5 QTL. These might include *FY*, *AGL15*, *CO*, *COL1*, *FRL1* and *LHP1* and *HUA2* (Table 1).

## Discussion

Arabidopsis accessions provide an excellent resource in which to explore the molecular basis of natural variation. We have been studying variation in flowering time and vernalization response between Arabidopsis accessions to address how Arabidopsis has adapted to growth in varying climates. In a previous study we undertook a preliminary QTL analysis on populations derived from four winter annual types, with very different vernalization responses crossed to the rapid cycler, Columbia. We established that the variation in vernalization response did not appear to map to any of the *trans*-factors currently known to mediate vernalization. Unexpectedly, despite the varying phenotypes of the parents most of the QTL mapped to very similar locations in the different populations. In this study we specifically aimed to further define these QTL by additional phenotypic analysis of the populations, both without vernalization and with longer, saturating, periods of vernalization. We also extended the analysis to include two N. American Arabidopsis accessions that showed interesting variation in flowering and *FLC* levels [6]. Our data indicate that just a few major QTL account for a large proportion of the flowering time variation in the six accessions analysed. The map positions of these QTL and their response to different vernalization periods suggest they may be caused by common loci. Smaller-effect QTL were also revealed particularly after saturating vernalization, which is consistent with a recent report based on genome wide association analysis of flowering time in natural accessions [40].

The common QTL corresponding to *FRI* on the top of chromosome 4 was expected given the use of Col-0, which carries a null *fri* allele, as the common parent. This QTL has been seen influencing flowering time in many other QTL studies [3], [5], [8], [11], [14], [22], [23], [25], [41], but here we show that it is also important in determining variation in vernalization response. Our data suggest that FRI activation of *FLC* still occurs even when expression of *FLC* has been partial silenced by vernalization and only when the vernalization requirement is fully saturated by extended periods of cold is FRI no longer able to influence *FLC* levels. This may reflect the difference between the silencing phase and the fully silenced state of the *FLC* locus [10].

Multiple QTL were resolved on chromosome 5 with a major effect QTL at the top of chromosome 5. This chromosomal region has been detected in QTL analyses of flowering time in many studies [5], [9], [14], [22], [23], [25]. Fine mapping of this region in recombinants from the Lov-1 × Col-0 population resolved at least two closely linked QTL in this region, one mapping near *FLC* (1.5–3.4 Mb) and a second between 4 and 6 Mb. Simon et al. (2008) also identified a number of candidates for a QTL at the top of chromosome 5 [25]. In two populations (Cvi × Col-0 and Shahdara × Col-0) a QTL close to *FLC* at 3.5 Mb was identified, while in a third population (Bur × Col) a QTL at 5.9 Mb was suggested to be *LHP1* [20]. Here we show that the QTL near *FLC* has differential affects after different vernalization treatments - a strong effect in plants that had not been vernalized, or had intermediate vernalization, and no significant effect after saturating (14 weeks of cold) vernalization. This is consistent with it being the result of *FLC* variation between the accessions and with our previous analysis, which found that epigenetic silencing of *FLC* quantitatively accumulated during the cold and the rate of this accumulation varied in different Arabidopsis accessions [10]. For Lov-1, Ull-2-5 and Var-2-6, a relatively short cold period (4 to 8 weeks) repressed *FLC* expression but the expression was reactivated when plants were returned to warm conditions. Their different behaviours suggest that although *FLC* is a common QTL the various Swedish accessions carry independent *FLC* alleles. The *FLC* alleles from Col-0, Lov-1, Ull-2-5 and Var-2-6 all encode the same protein so allelic variation is likely to represent changed expression, consistent with our previous study [10]. In a genome wide comparison of polymorphisms Ull-2-5 and Var-2-6 are two of the most dissimilar accessions from southern Sweden and they group independently from the N. Swedish Lov-1 accession [36], consistent with them carrying independent *FLC* alleles. Recent evidence supports the presence of rare alleles of large effect influencing flowering time in Arabidopsis accessions [42].

*FLC* also emerged as a QTL for flowering time and vernalization in populations derived from the two N. American accessions Kno-18 and RRS-10 crossed to Col. Kno-18 and RRS-10 share an identical *FLC* allele with a low expression level and a relative insensitivity to vernalization. These effects appear to be caused by insertion of a 1.19 kb Mutator-like transposable element insertion in intron 1, as judged by the experiments inserting the transposable element into the Col allele. The allelic effect of the QTL switches in individuals homozygous for this *FLC* allele; non-vernalized, they flower earlier than those carrying the Col allele, and later once they have been vernalized. The transposon insertion appears, therefore, to attenuate pathways that up-regulate *FLC* before vernalization and silence *FLC* during vernalization. On this basis, the small phenotypic variation between Kno-18 and RRS-10 would appear to be the result of other genes very closely linked to *FLC* and to the QTL on chromosomes 1 and 4.

Five other N. American accessions (but no accession outside N. America) share the 1.19 kb Mutator-like transposable element insertion (*FLC^TE490^*) at the 5′ end of intron 1 and they show similar phenotypic flowering behaviour. Other Arabidopsis accessions also have transposon insertions in intron 1 of *FLC*, and these generally give rise to weak alleles [39], [41], [43]. Indeed, TE490 is almost identical to the insertion found at the 3′ end of intron 1 in the L*er FLC* allele, a weak allele caused by siRNAs generated from homologous copies of the TE directing H3K9 methylation to the *FLC* locus and reducing its transcription [39], [44]. A similar mechanism may arise in the *FLC^TE490^* allele given the homology to the other endogenous elements.

A clear candidate for the QTL at ∼24Mb on chromosome 1 is *FT* (24.3 Mb). QTL for flowering time have been previously mapped to this region [23] [14], [25], and Schwartz *et al* [15] recently showed that allelic variation in a 6.7 kb fragment in the *FT* promoter leads to expression polymorphism and flowering time variation. In this study, the refinement of the QTL on chromosome 1 in the Ull-2-5 × Col-0 population followed by fine-mapping pointed to polymorphism in a similar genomic region resulting in allelic variation at *FT*. The sequences of *FT* regulatory region in Ull-2-5 and Est-1 are very different, but they both caused impaired *FT* expression pattern in response to long day induction. The interval mapped in Est-1 and Ull2-5 contains the functional block B and C identified by Adrain et al in the *FT* promoter region [45]. We compared these blocks in Est-1 and Ull2-5, and found no difference between them for the block B sequence, but within block C, which might contain crucial elements required for the response to CONSTANS [45], we found one polymorphism in the CCAAT box in Ull2-5, but not in Est-1 (Table S3). We now need to determine the exact causative variation in these two unrelated accessions, and to determine how *FT* expression is altered. The variation in the *FT cis*-regulatory regions is yet another pertinent example in the debate over the importance of regulatory and coding sequence variation in evolution. There is increasing evidence to show that mutations within *cis*-regulatory regions underlie a variety of interesting and ecologically significant phenotypic differences [46] and the observed variation at *FT* supports this view. Since *FT* is the target of many different flowering pathways [2] variation affecting protein function would constitutively influence flowering time. In contrast, variation in regulatory regions could lead to a specific adaptation to one type of environmental cue via the tuning of *FT* activation through just one pathway. Analysis of the Col-0 genomic sequence revealed that the gene density around *FT* region is very unusual for Arabidopsis; *FT* is the only gene within a 20 kb region. Given the number of pathways regulating *FT* this region may contain many regulatory *cis*-elements; indeed this unusual pattern of genome organization may be the result of the accumulation of regulatory sequences which control a central regulator of a major adaptive trait, i.e. flowering. It will be important in the future to identify the exact causative polymorphism to further understand its role in *FT* evolution.

The variation in the Ull-2-5 *FT* region makes flowering in the Ull-2-5 accession less sensitive to long day induction compared to the Col-0 genotype. This effect is most strongly revealed after 14 weeks of vernalization suggesting that in Sweden this variation is most important in spring, after the vernalization requirement has been satisfied. The Ull-2-5 accession was collected form disturbed ground in a meadow, a more competitive habitat than is generally envisaged as the typical Arabidopsis niche, thought to be open, disturbed ground. The delayed flowering would intuitively seem to provide a fitness advantage in this more competitive habitat through extension of vegetative development leading to larger more robust plants with high seed yield. We need to combine ecological analyses with our molecular dissection to test these ideas.

## Materials and Methods

### Construction of mapping populations

Arabidopsis accessions Lov -1, Ull-2-5, Var-2-6, and Edi-0 were crossed to Col-0 and the resulting F1 plants allowed to self; 184 F2 lines per population were generated [10]. F2 seeds were sown on soil in plastic pots (7 cm×7 cm) and stratified at 5°C with an 8 hour photoperiod and constant humidity (70%) for 3 days. Seeds were moved to a growth room at 23°C, with a 16 hour photoperiod, for 7 days to allow germination and pre-growth. The seedlings were then transferred back into 5°C for a treatment of either 4 weeks (Lov-1× Col-0 and Edi-0 × Col-0) or 8 weeks (Var-2-6 × Col-0 and Ull-2-5 × Col-0). After vernalization, F2 seedlings were transplanted into trays with 40 cells of 2 cm×2 cm and moved back to 23°C with a 16 hour photoperiod. Trays were moved regularly to random positions to prevent any positional effects on plant growth. For the subsequent re-phenotypic analysis of the population 50 % of the F2 lines were chosen at random and 20 F3 seed from each F2 line were grown as described above, without vernalization (Lov-1 × Col-0, Edi-0 × Col-0) or after 14 weeks of vernalization (Lov-1 × Col-0, Ull-2-5 × Col-0). Plants were transplanted in a semi-random manner, and trays were randomised within the growth room.

### Phenotypic data collection

Flowering time was scored as either total leaf number (rosette leaves plus cauline leaves at flowering) or bolting time. The number of leaves was counted to a maximum of 150 so individuals that had not flowered were given the value 150. For bolting time the number of days-to-flowering as scored when the inflorescence stem reached 3 cm. The mean and standard error of up to 20 plants per line was calculated.

### Marker scoring

SNP markers were designed (from the SNP information generated in the laboratory of Magnus Nordborg, USC, USA), screened and verified at the MPI, Tuebingen. In total 56 - 59 markers distributed across the five chromosomes with an average distance of 2–3 Mb and near to possible candidate flowering time genes were chosen [47] . DNAs from all F2 plants were genotyped for these SNP markers by Genaissance Pharmaceuticals Inc (New Haven, CT). *FRI* was scored on the populations using primers spanning the 16 bp deletion in Col-0. Parental F2 genotyping data was also used for the F3 phenotypic analysis.

### Genetic mapping and QTL analysis

Segregation analysis was performed and the linkage map generated using MAPMAKER version 3.0 b [48]. The recombination fractions were converted to centiMorgans (cM) using the Kosambi mapping function. Marker segregation distortion was calculated using Windows QTL Cartographer version 2.5 chi^2^ test result, at 0.1 % significance level. Markers that had a high failure rate were discarded in the segregation analysis as they were likely to show distortion for technical rather than biological reasons. Trait data was assessed for normality in Genstat version 10.1.

The QTL analysis was performed with Windows QTL Cartographer version 2.5 using Composite Interval Mapping (CIM) method with Model 6: Standard model. Cofactors were identified with forward and reverse regression, the window size was set at 5.0 cM, the walk speed at 2.0 cM and the probability for into or out of set at 0.05. The threshold for significance was calculated by 1000 permutations test for 0.05 probability.

The effect of the QTL and the variance they accounted for was calculated in QTL Cartographer version 2.5 using Multiple Interval Mapping (MIM). QTL found in the CIM model were entered into the MIM model, which then identified the effect of the QTL. The percentage variance explained by the QTL is the R^2^ value multiplied by 100.

### RNA extraction and real-time quantitative PCR analysis

Total RNA was prepared and first strand cDNA was synthesized using Invitrogen Reverse Transcription kit (No. 12371-09) according to manufacturer's instructions. Real-time Quantitative PCR was performed using Sigma SYBR Green Jumpstart kit (No. S4438). Primers for the *UBC* internal control for *FT* expression analysis are: Forward: 5′-CTGCGACTCAGGGAATCTTCTAA-3′ and Reverse: 5′-TTGTGCCATTGAATTGAACCC-3′; Primers for *FT* are: Forward: 5′-CTGGAACAACCTTTGGCAAT-3′ and Reverse: 5′-AGCCACTCTCCCTCTGACAA-3′. Normal RT-PCR was applied to analyse the expression of *FAS1*. Primers for *FAS1* are: Forward: 5′-CTTCCCATTCTTCATCACTATCAACTTC-3′ and Reverse: 5′-TGTTCAGGCAATTGACAACGC-3′. *UBQ10* was used as internal control as described before [49].

### FLC^TE490^ transformant lines

The *FLC* transposon from Kno-18 was amplified by PCR and cloned into pENT-ColFLC plasmid using SapI/BsgI, known as pENT FLC^ TE490^. The Gateway® recombination system was used to recombine pENT-FLC^TE490^ and pDEST-SLJ to create the final pDEST-SLJ-FLC^TE490^ construct. The construct was transformed into *E*. *coli* and transferred to *Agrobacterium tumefaciens* by a tri-parental mating. Col *FRI* Sf2 *flc*-2 and Col *FRI* Sf2 *flc*-3 plants were grown to flowering and transformed using the floral dip transformation protocol with the *Agrobacterium* containing the pDEST-SLJ-FLC^TE490^ construct. T1 lines were selected by BASTA spraying and homozygous T3 plants were used for the final experiments.

## Supporting Information

Figure S1 **Histograms showing flowering time of different populations.** The flowering time is shown on the x-axis as days-to-flower (F3 populations) or final leaf number (F2 populations), and number of individuals on the y-axis. The parental accessions and average of the F2 or F3 progeny are shown by arrows. (A) Lov-1 x Columbia (B) Ull-2-5 x Columbia (C) Var-2-6 x Columbia (D) Edi-0 x Columbia (E) Kno-18 x Columbia (F) RRS-10 x Columbia.
(TIF)

Figure S2 **Genetic map of the 6 populations showing markers used in the QTL analysis.** Markers with segregation distortion at 0.1% significance level are marked with asterix, segregation bias towards Columbia *, segregation bias towards other parent **. A) Lov-1 x Col, B) Ull-2-5 x Col, C) Var-2-6 x Col, D) Edi-O x Col, E) Kno-18 x Col, F) RRS-10 x Col.
(TIF)

Figure S3 **Average flowering time of the QTL populations grouped into genotype classes.**
(TIF)

Figure S4 **Flowering time of specific genotypes from inbred lines generated from backcrossing Lov-1 × Col-0 plants to Col** ***FRI*** **.**
(TIF)

Figure S5 **Flowering time of Kno-18 × Col-0 F2 population without vernalization grouped by genotype at marker underlying the QTL.**
(TIF)

Figure S6 **Flowering time of RRS-10 × Col F2 population after 8 weeks vernalization grouped by genotype at marker underlying the QTL.**
(TIF)

Figure S7 **Polymorphisms within** ***FLC*** **genomic fragment of Kno-18 and RRS-10.**
(TIF)

Figure S8 ***FLC^TE490^* transposon insertion in RRS-10 and Kno-18.**
(TIF)

Table S1 **Markers and their alternative names used to generate the genetic maps.**
(XLSX)

Table S2 **Primer sequences of mapping markers developed in this study.**
(DOC)

Table S3 **Alignment of** ***FT*** **regulatory sequences of Col-0, Ull-2-5 and Est-1.**
(DOC)
