# Supplementary figures and images for: Major-Effect Alleles at Relatively Few Loci Underlie Distinct Vernalization and Flowering Variation in Arabidopsis Accessions
Source: PLoS One. 2011 May 20;6(5):e19949. doi: 10.1371/journal.pone.0019949 (PMC3098857; doi:10.1371/journal.pone.0019949)

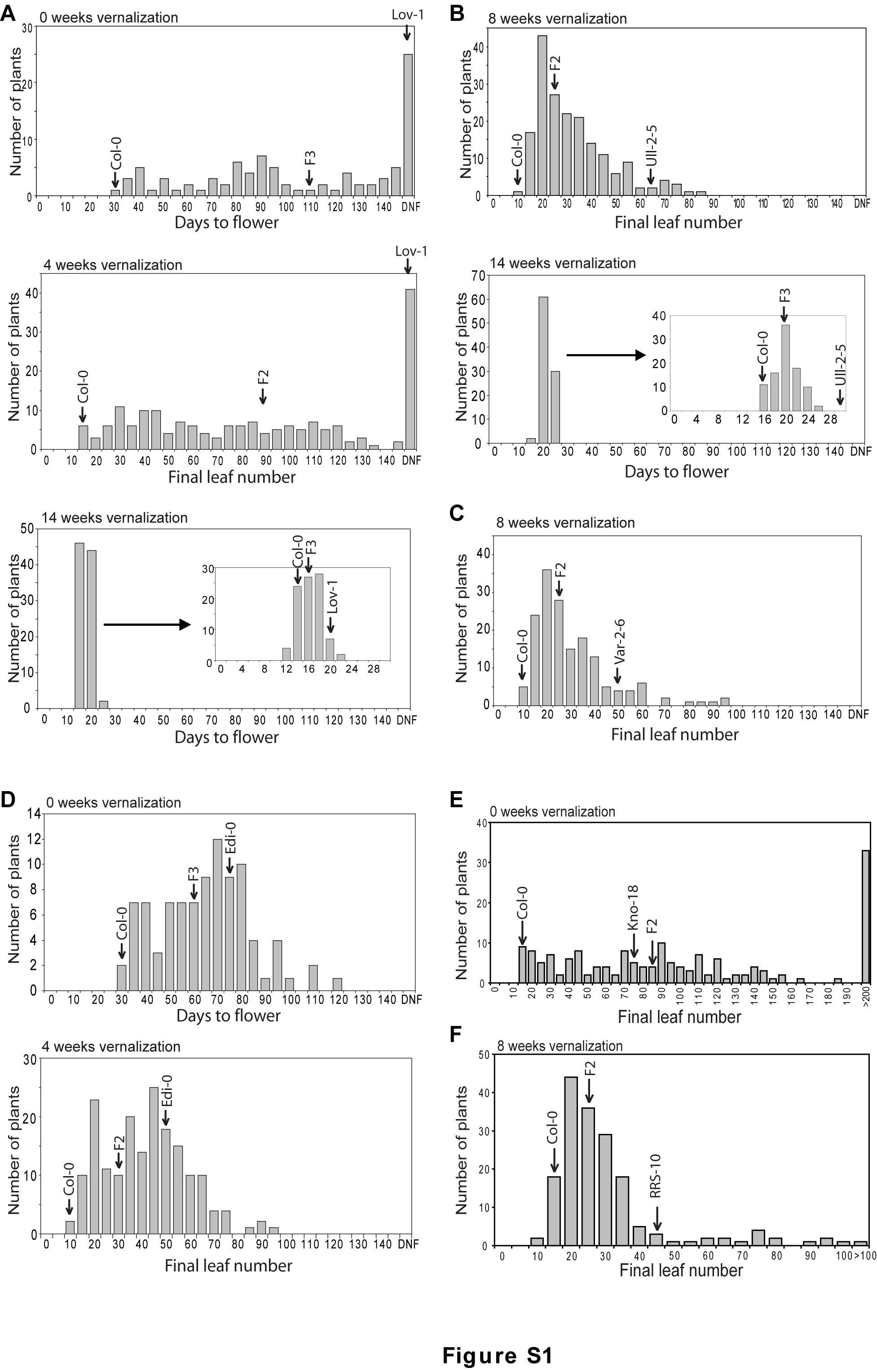

Supplement: Figure S1 — Histograms showing flowering time of different populations. The flowering time is shown on the x-axis as days-to-flower (F3 populations) or final leaf number (F2 populations), and number of individuals on the y-axis. The parental accessions and average of the F2 or F3 progeny are shown by arrows. (A) Lov-1 x Columbia (B) Ull-2-5 x Columbia (C) Var-2-6 x Columbia (D) Edi-0 x Columbia (E) Kno-18 x Columbia (F) RRS-10 x Columbia. (TIF) [file pone.0019949.s001.tif]

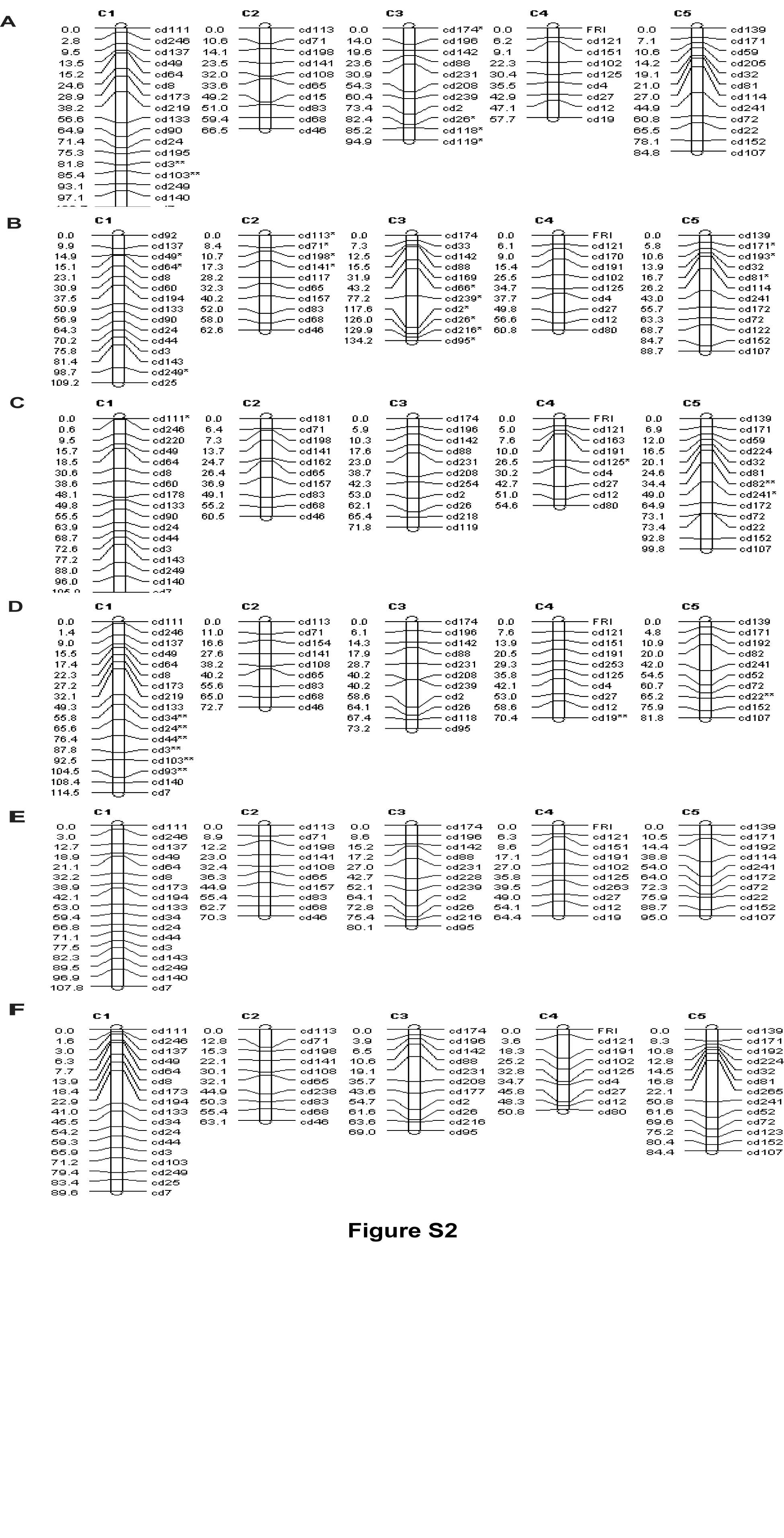

Supplement: Figure S2 — Genetic map of the 6 populations showing markers used in the QTL analysis. Markers with segregation distortion at 0.1% significance level are marked with asterix, segregation bias towards Columbia *, segregation bias towards other parent **. A) Lov-1 x Col, B) Ull-2-5 x Col, C) Var-2-6 x Col, D) Edi-O x Col, E) Kno-18 x Col, F) RRS-10 x Col. (TIF) [file pone.0019949.s002.tif]

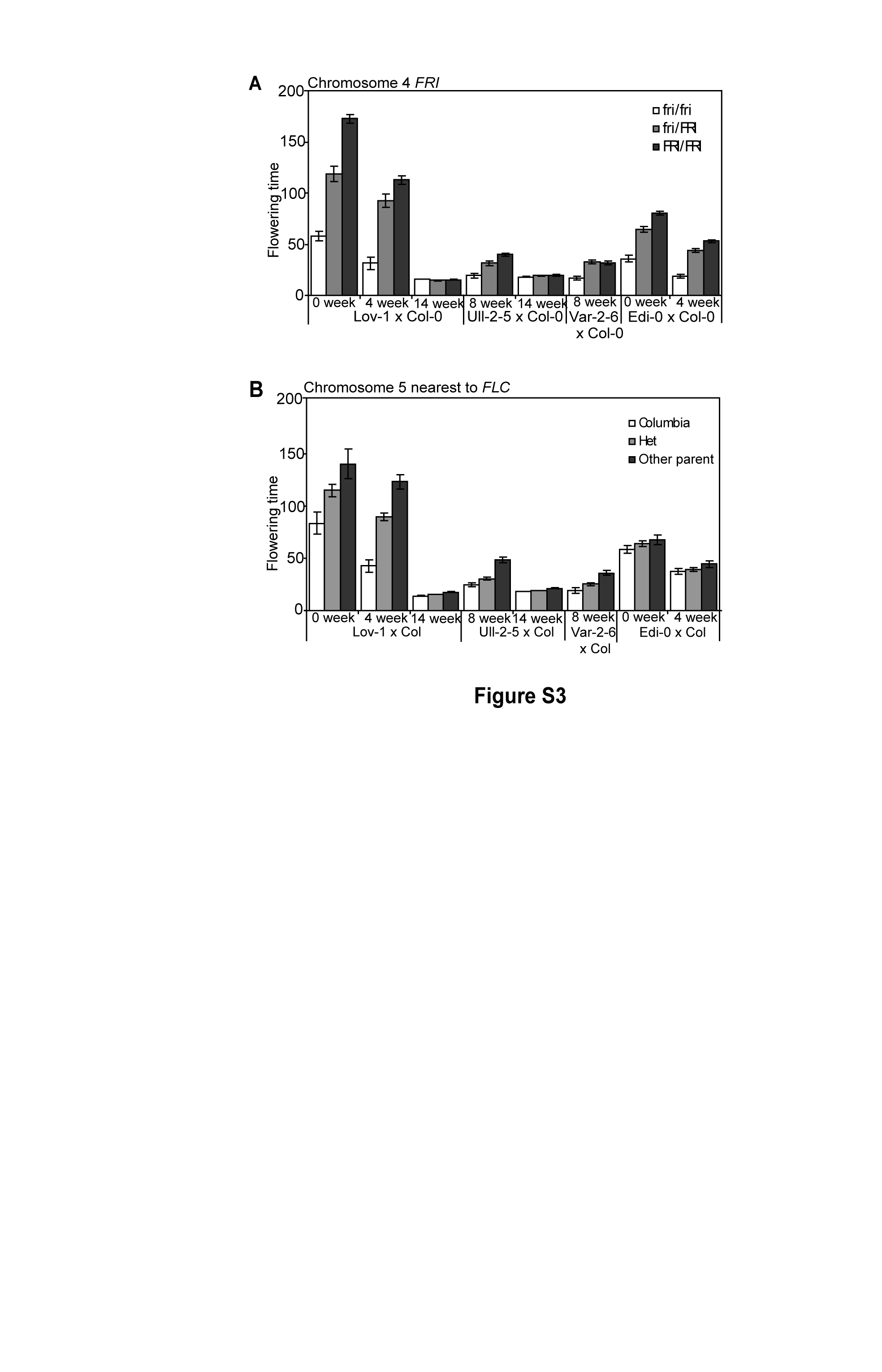

Supplement: Figure S3 — Average flowering time of the QTL populations grouped into genotype classes. (TIF) [file pone.0019949.s003.tif]

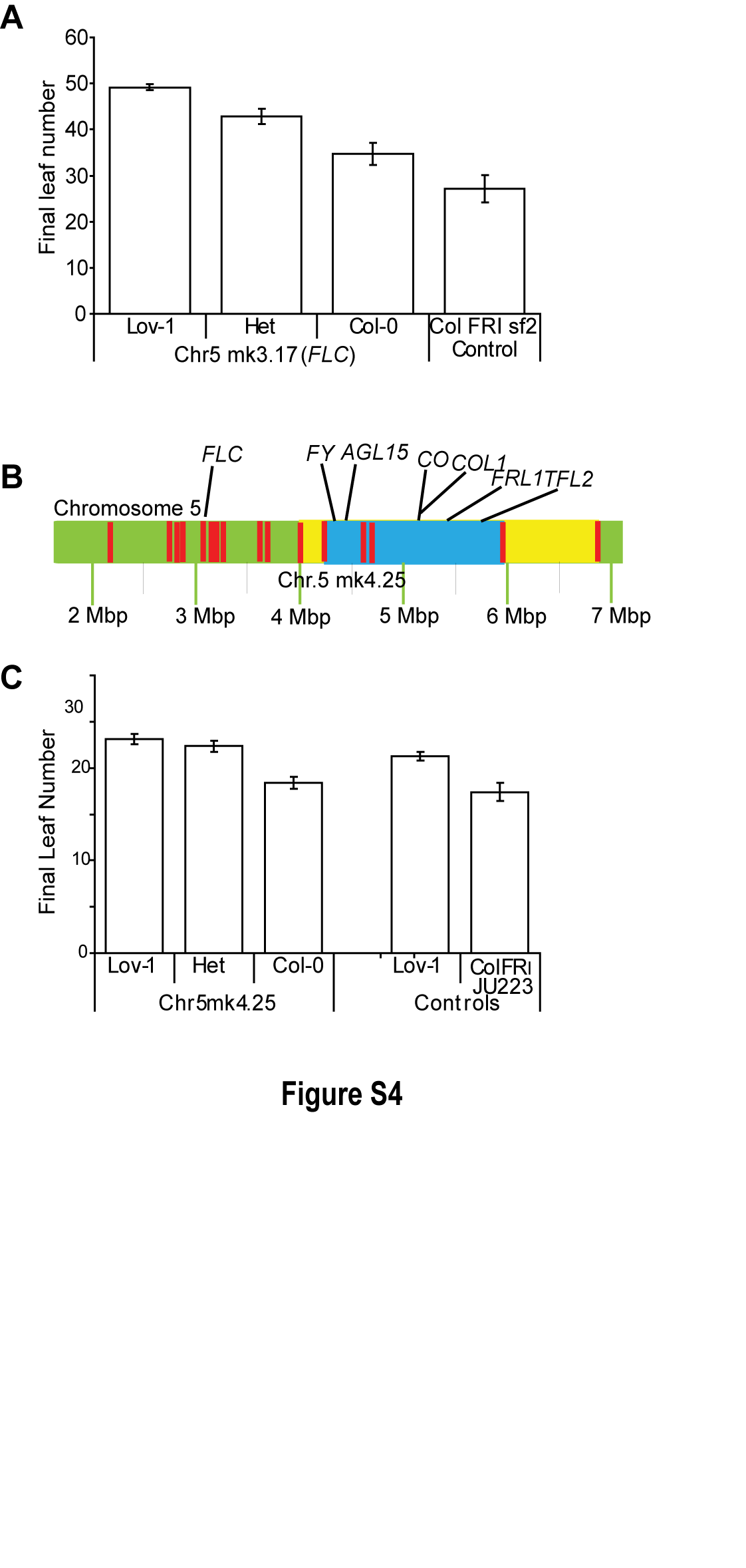

Supplement: Figure S4 — Flowering time of specific genotypes from inbred lines generated from backcrossing Lov-1 × Col-0 plants to Col FRI . (TIF) [file pone.0019949.s004.tif]

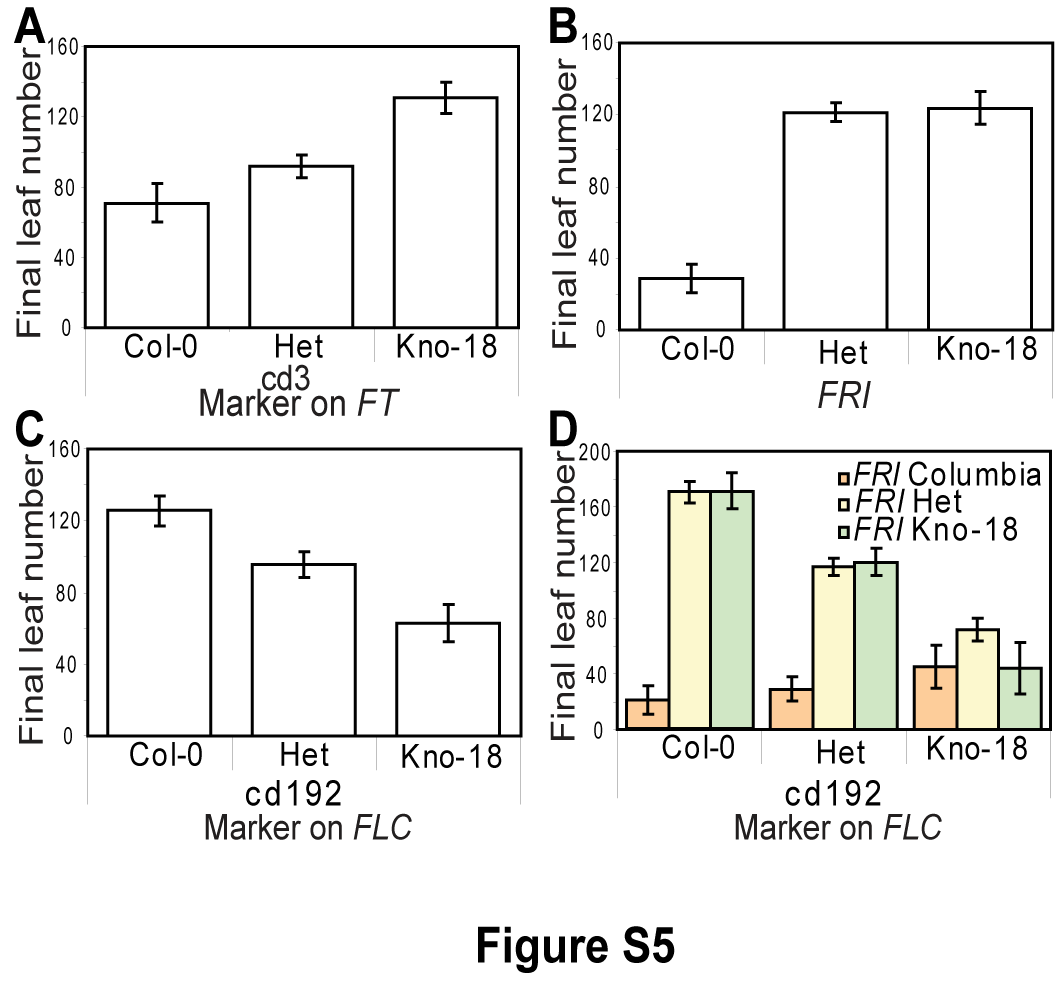

Supplement: Figure S5 — Flowering time of Kno-18 × Col-0 F2 population without vernalization grouped by genotype at marker underlying the QTL. (TIF) [file pone.0019949.s005.tif]

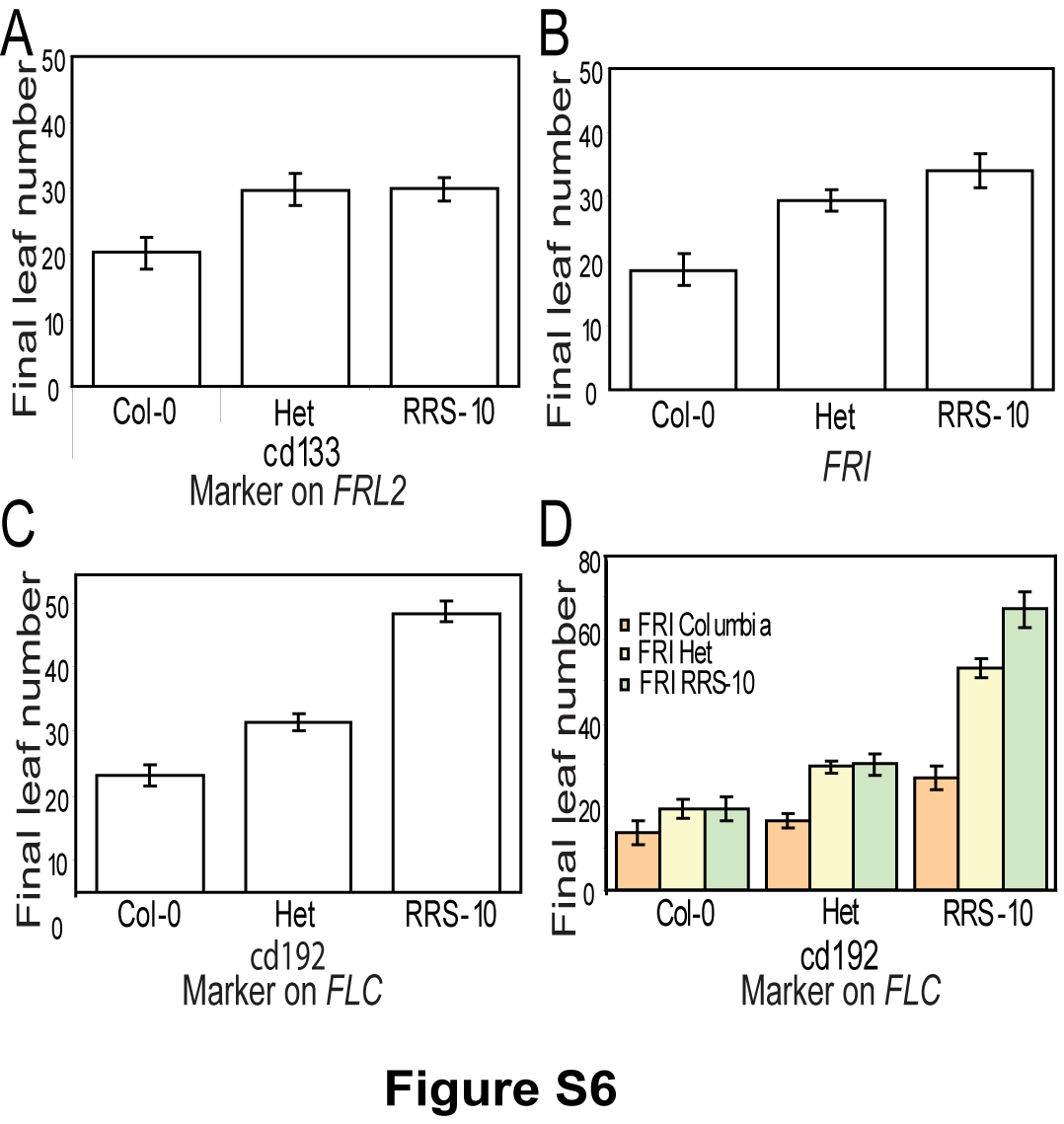

Supplement: Figure S6 — Flowering time of RRS-10 × Col F2 population after 8 weeks vernalization grouped by genotype at marker underlying the QTL. (TIF) [file pone.0019949.s006.tif]

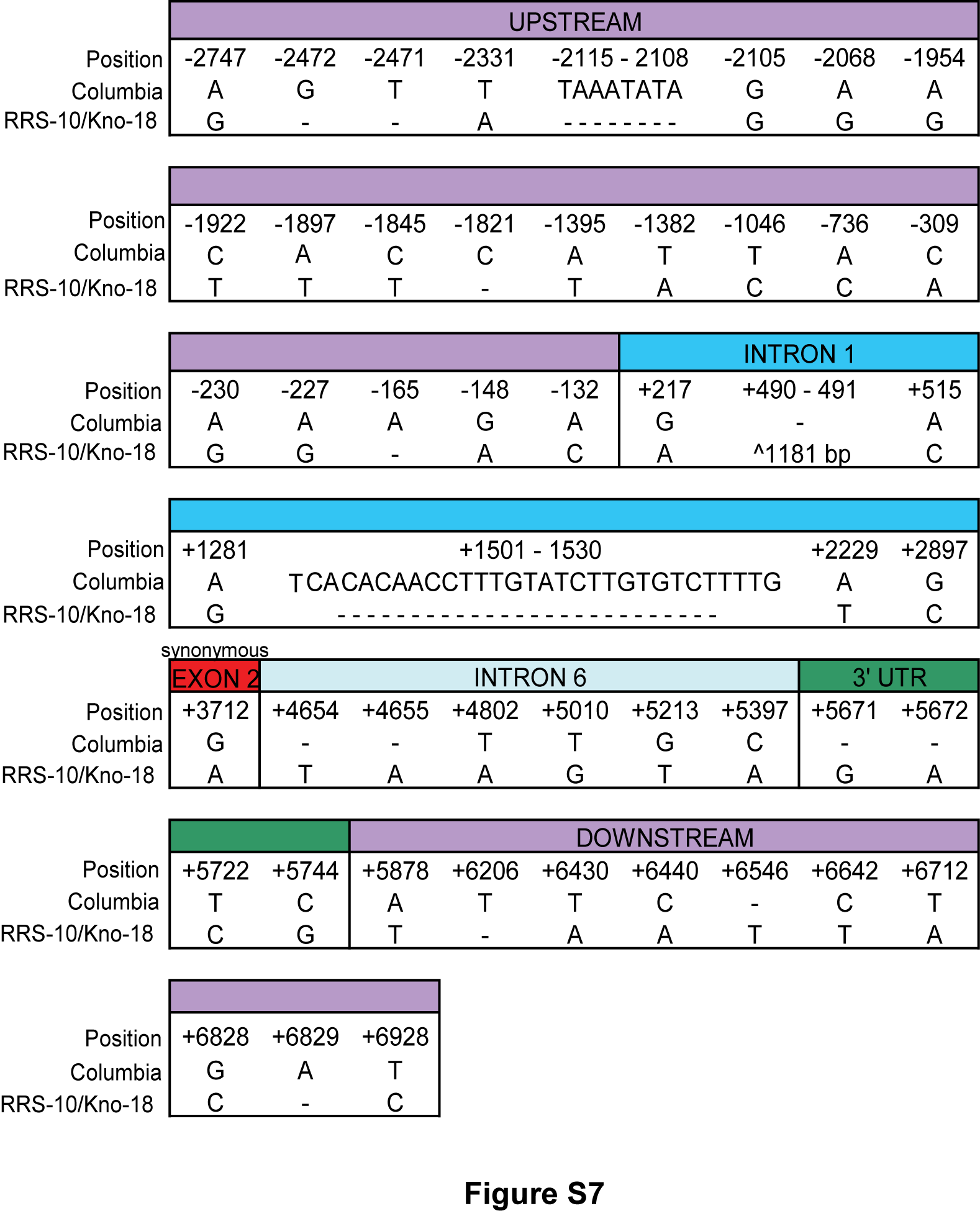

Supplement: Figure S7 — Polymorphisms within FLC genomic fragment of Kno-18 and RRS-10. (TIF) [file pone.0019949.s007.tif]

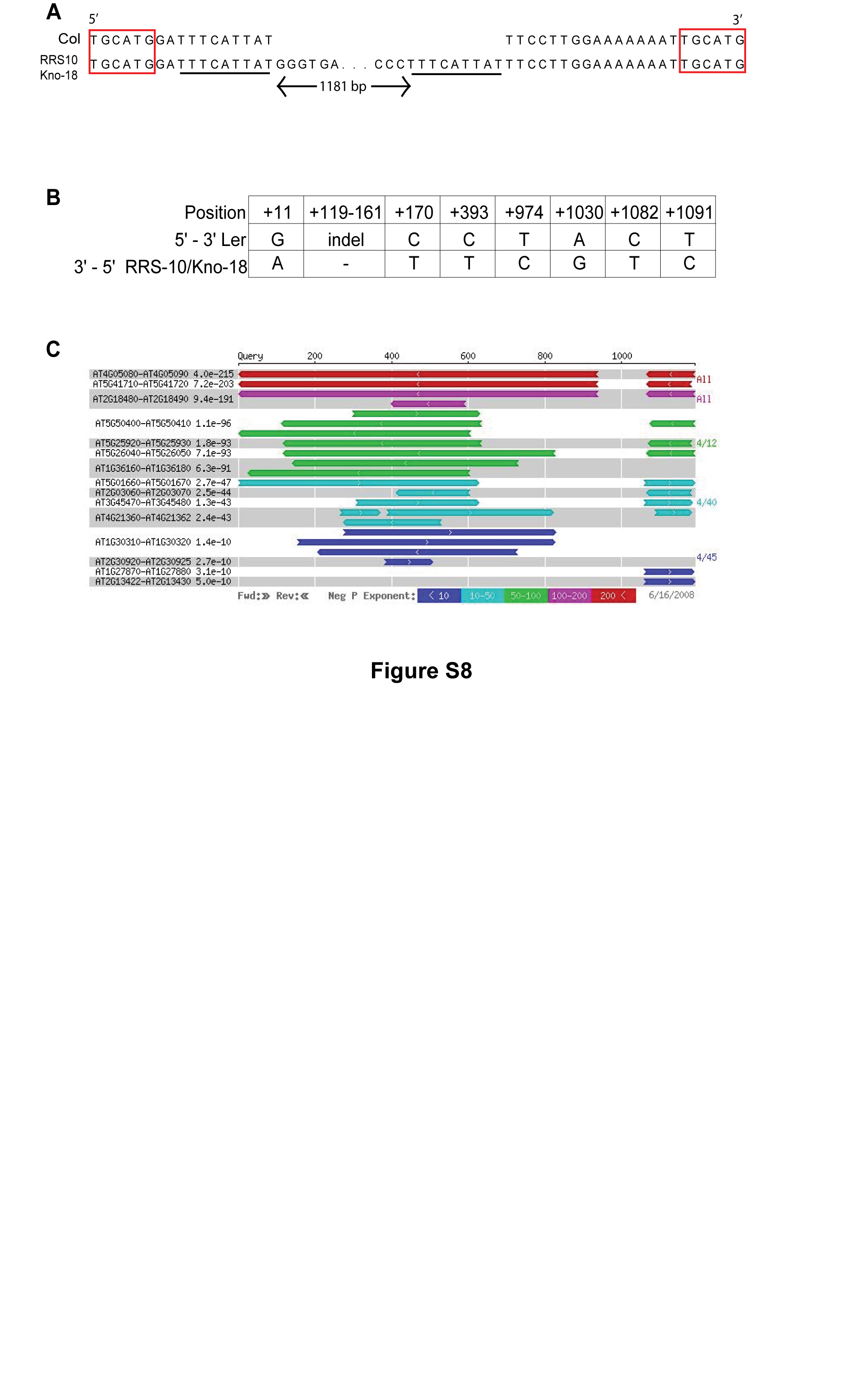

Supplement: Figure S8 — FLCTE490 transposon insertion in RRS-10 and Kno-18. (TIF) [file pone.0019949.s008.tif]
