# Supplementary material for: Major-Effect Alleles at Relatively Few Loci Underlie Distinct Vernalization and Flowering Variation in Arabidopsis Accessions
Source: PLoS One. 2011 May 20;6(5):e19949. doi: 10.1371/journal.pone.0019949 (PMC3098857; doi:10.1371/journal.pone.0019949)
Supplement: Table S2 — Primer sequences of mapping markers developed in this study. (DOC) [file pone.0019949.s010.doc]

| **Primer name** | **Forward** | **Reverse** | **Marker type** |
| --- | --- | --- | --- |
| Chr.5 mk3.17 | TATATGCACGTCCGGGAGAT | GAGGCACCAAAGAAACAAGG | SSLP |
| Chr.5 mk4.25 | CCCAGTCTAACCACGACCAC | AATCCCAGTAACCAAACACACA | SSLP |
| FRL2 | AGCTTCCTAATGCGATTCGAT | CCAAAACGAAGATCCTCTTCA | - |
| L24 | ATCTACAGGTTCCCAATCAGC | CTGGATGTGTCGTACTTGTTCAC | dCAPS/AluI |
| L2432 | TAACGGATGAGTATGCGCGAGC | CTATACTTAGGCTTTCCCCAAG | dCAPs/AluI |
| CAF5 | CAGAACAGGGTAAAGTAGCG | AGTGGAGTAACATCTGACGGTG | STS |
| 12INS | CGTTTCCTTTATCTACATTACC | TATCAACTCCACAAATAGCATC | STS |
| FT28 | CTCTATAAACTTGGCGGTACCC | TCTCCCACTTGGTAGCCACT | STS |
| R24468 | AATGAACCAAAACCAAAGAGAGC | CTGGAGAAATGTATGGACCCTAC | dCAPs/AluI |
| R24 | ATTCCGCTTTAGACGAGATAAGC | CATACTCTTCGCCTATACTCATATC | dCAPs/AluI |
| 538D | AATAACTTGTTTTCGGACTTGG | CGAAGATTGGCAAGTGGATG | STS |

Table S2 Primer sequences for mapping markers developed in this study
