# Supplementary material for: Major-Effect Alleles at Relatively Few Loci Underlie Distinct Vernalization and Flowering Variation in Arabidopsis Accessions
Source: PLoS One. 2011 May 20;6(5):e19949. doi: 10.1371/journal.pone.0019949 (PMC3098857; doi:10.1371/journal.pone.0019949)
Supplement: Table S3 — Alignment of FT regulatory sequences of Col-0, Ull-2-5 and Est-1. (DOC) [file pone.0019949.s011.doc]

**Table S4** **Alignment of *FT* regulatory sequences of Col-0, Ull-2-5 and Est-1**

-7972*

....|....|....|....|....|....|....|....|....|....|....|....|....|....|....|....|....|....|....|....|

**Col-0 FT**  **CCATAGTAACACTTTCAGTCTTCTCCTTCAATTTCAAGTAAATCTCATCTACAAGCTTCGACAACGGCAAACTCATTTCCTCCATTAACAAAGCCACCAT**

**Ull-2-5 FT** **CCATAGTAACACTTTCAGTCTTCTCCTTCAATTTCAAGTAAATCTCATCTACAAGCTTCGACAACGGCAAACTCATTTCCTCCATTAACAAAGCCACCAT**

**Est-1 FT**  **CCATAGTAACACTTTCAGTCTTCTCCTTCAATTTCAAGTAAATCTCATCTACAAGCTTCGACAACGGCAAACTCATTTCCTCCATTAACAAAGCCACCAT**

-7872

....|....|....|....|....|....|....|....|....|....|....|....|....|....|....|....|....|....|....|....|

**Col-0 FT**  **CGAATTCAATGAGGAACATTCACTAAATCCAGAAAACAGATCTGTTCTCTTACTCTTATCCATAACTTCTCTGAAATAATCAAAAAGCCCTTTCATCTCT**

**Ull-2-5 FT** **CGAATTCAATGAGGAACATTCACTAAATCCAGAAAACAGATCTGTTCTCTTACTCTTATCCATAACTTCTCTGAAATAATCAAAAAGCCCTTTCATCTCT**

**Est-1 FT**  **CGAATTCAATGAGGAACATTCACTAAATCCAGAAAACAGATCTGTTCTCTTACTCTTATCCATAACTTCTCTGAAATAATCAAAAAGCCCTTTCATCTCT**

-7772

....|....|....|....|....|....|....|....|....|....|....|....|....|....|....|....|....|....|....|....|

**Col-0 FT**  **AGATTCAACGACGAGATCTGAGATTCTTTCTCCTCAGATGTTAGATTCTCAATCGCCGTGGGTTCTCTCTTCCGCTTATTCAGCTTCTTTGGCTCTATCA**

**Ull-2-5 FT** **AGATTCAACGACGAGATCTGAGATTCTTTCTCCTCAGATGTTAGATTCTCAATCGCCGTGGGTTCTCTCTTCCGCTTATTCAGCTTCTTTGGCTCTATCA**

**Est-1 FT**  **AGATTCAACGACGAGATCTGAGATTCTTTCTCCTCAGATGTTAGATTCTCAATCGCCGTGGGTTCTCTCTTCCGCTTATTCAGCTTCTTTGGCTCTATCA**

-7672

....|....|....|....|....|....|....|....|....|....|....|....|....|....|....|....|....|....|....|....|

**Col-0 FT**  **TCGTCTTCCGATTCTCATTTTCATTCACCGTCGAAACTTCGTCCATCGCAAAAAAAAAAA---CTAGAAAAATTCGGGAATCTATAAACCCAGGAAAATT**

**Ull-2-5 FT** **TCGTCTTCCGATTCTCATTTTCATTCACCGTCGAAACTTCGTCCATCGCAAAAAAAAAAAAAACTAGAAAAATTCGGGAATCTATAAACCCAGGAAAATT**

**Est-1 FT**  **TCGTCTTCCGATTCTCATTTTCATTCACCGTCGAAACTTCGTCCATCGCAAAAAAAAAAAAA-CTAGAAAAATTCGGGAATCTATAAACCCAGGAAAATT**

-7575

....|....|....|....|....|....|....|....|....|....|....|....|....|....|....|....|....|....|....|....|

**Col-0 FT**  **GAAACAAGATCTAGAATCTAAGAAATCGATTGCGAGAAACACGAGCTGAGTGGAGATTTGTTGTTCCTTTCGTGATTTTGATTTTGGATTTTTTTTTTCT**

**Ull-2-5 FT** **GAAACAAGATCTAGAATCTAAGAAATCGATTGCGAGAAACACGAGCTGAGTGGAGATTTGTTGTTCCTTTCGTGATTTTGATTTTGGATTTTTTTTTTCT**

**Est-1 FT**  **GAAACAAGATCTAGAATCTAAGAAATCGATTGCGAGAAACACGAGCTGAGTGGAGATTTGTTGTTCCTTTCGTGATTTTGATTTTGGATTTTTTTTTTCT**

-7475

....|....|....|....|....|....|....|....|....|....|....|....|....|....|....|....|....|....|....|....|

**Col-0 FT**  **CAGAGTTCTTAGGTTTTTGGAGAAGAAGAAGAAGAAGGGAGTGGGAAGATGGATAGGTTTGAAAAAAAAAAAGGATAAAAAAATAAAATTGGGGATTCAC**

**Ull-2-5 FT** **CAGAGTTCTTAGGTTTTTGGAGAAGAAGAAGAAGAAGGGAGTGGGAAGATGGATAGGTTTGAAAACAAAAAGGGATAAAAAA-TAAAATTGGGGATTCAC**

**Est-1 FT**  **CAGAGTTCTTAGGTTTTTGGAGAAGAAGAAGAAGAAGGGAGTGGGAAGATGGATAGGTTTGAAAAAAAAAA-GGATAAAAAAATAAAATTGGGGATTCAC**

-7375

....|....|....|....|....|....|....|....|....|....|....|....|....|....|....|....|....|....|....|....|

**Col-0 FT**  **TAAAAAGAAGGGTCGTTGATTTTCCCGCCAAGGTTTTGACGGGTGGATTTGCCTCCAAATGACAAAAATGCAATTTTACTGTTTGGCGCCACTCAAGTTT**

**Ull-2-5 FT** **TAAAAAGAAGGGTCGTTGATTTTCCCGCCAAGGTTTTGACGGGTGGATTTGCCTCCAAATGACAAAAATGCAATTTTACTGTTTGGCGCCACTTAAGTTT**

**Est-1 FT**  **TAAAAAGAAGGGTCGTTGATTTTCCCGCCAAGGTTTTGACGGGTGGATTTGCCTCCAAATGACAAAAATGCAATTTTACTGTTTGGCGCCACTCAAGTTT**

-7275

....|....|....|....|....|....|....|....|....|....|....|....|....|....|....|....|....|....|....|....|

**Col-0 FT**  **TGGAGAAGATGAAGGCCTATGTGGGCCTGCTTTTGTTTCTTTTAAGCCCAATATAGTGGGGGAAACAATTGGGATGACATATAAATAGAGACTAGAATTT**

**Ull-2-5 FT** **TGGAGAAGATGAAGGCCTATGTGGGCCTGCTTTTGTTTCTTTTAAGCCCAAAATAGTGGGGGAAACAATTGGGATGACATATAAATAGAGACTAGAATTT**

**Est-1 FT**  **TGGAGAAGATGAAGGCCTATGTGGGCCTGCTTTTGTTTCTTTTAAGCCCAATATAGTGGGGGAAACAATTGGGATGACATATAAATAGAGACTAGAATTT**

-7175

....|....|....|....|....|....|....|....|....|....|....|....|....|....|....|....|....|....|....|....|

**Col-0 FT**  **ACTAATTTTAAATACTCATGCGGATGCGGTTATATAAATAGAGACTAGAAATTACTCATTTTAATTTTAATTTTAAGCCTAATACGGTCATGGTGAGCAA**

**Ull-2-5 FT** **ACTAATTTTAAATACTCATGCGGATGCGGTTATATAAATAGAGACTAGAAATTACTCATTTTAATTTTAATTTTAAGCCTAATACGGTCATGGTGAGCAA**

**Est-1 FT**  **ACTAATTTTAAATACTCATGCGGATGCGGTTATATAAATAGAGACTAGAAATTACTCATTTTAATTTTAATTTTAAGCCTAATACGGTCATGGTGAGCAA**

-7075

....|....|....|....|....|....|....|....|....|....|....|....|....|....|....|....|....|....|....|....|

**Col-0 FT**  **GAGCTCCGAATGAAACCGAATAATACTTTGGATACGATTTATGAACGAGGTAAGAAAGTGAAAGGAAATGAAGATTTGTAGCAAGAAAAGGGAGGGAGAG**

**Ull-2-5 FT** **GAGCTCCGAATGAAACCGAATAATACTTTGGATACGATTTATGAACGAGGTAAGAAAGTGAAAGGAAATGAAGATTTCTAGCAAGAAAAGGGAGGGAGAG**

**Est-1 FT**  **GAGCTCCGAATGAAACCGAATAATACTTTGGATACGATTTATGAACGAGGTAAGAAAGTGAAAGGAAATGAAGATTTGTAGCAAGAAAAGGGAGGGAGAG**

-6975

....|....|....|....|....|....|....|....|....|....|....|....|....|....|....|....|....|....|....|....|

**Col-0 FT**  **AAGATGGTGAGGTTTTTGTTCACTGAGCATATGTTGTTGACTGACCATATGAGGGAATCAATTGATGATGAATGATGTGGTAGTAGTGCGTGTTAATTTG**

**Ull-2-5 FT** **AAGATGGTGAGGTTTTTGTTCACTGAGCATATGTTGTTGACTGACCATATGAGGGAATCAATTGATGATGAATGATGTGGTAGTAGTGCGTGTTAATTTG**

**Est-1 FT**  **AAGATGGTGAGGTTTTTGTTCACTGAGCATATGTTGTTGACTGACCATATGAGGGAATCAATTGATGATGAATGATGTGGTAGTAGTGCGTGTTAATTTG**

-6875

....|....|....|....|....|....|....|....|....|....|....|....|....|....|....|....|....|....|....|....|

**Col-0 FT**  **TCGTTAGAAAAATGTTTGAATTATAATTCTTCGCTTTAATTTTCTTCTAAAAGATACTAACAGTCATACTAATTAAGAATGAGTAATTGGTTGGACAATT**

**Ull-2-5 FT** **TCGTTAGAAAAATGTTTGAATTATAATTCTTCGCTTTAATTTTCTTCTAAAAGATACTAACAGTCATACTAATTAAGAATGAGTAATTGGTTGGACAATT**

**Est-1 FT**  **TCGTTAGAAAAATGTTTGAATTATAATTCTTCGCTTTAATTTTCTTCTAAAAGATACTAACAGTCATACTAATTAAGAATGAGTAATTGGTTGGACAATT**

-6775

....|....|....|....|....|....|....|....|....|....|....|....|....|....|....|....|....|....|....|....|

**Col-0 FT**  **CAATAAGCAAACAAAGTGAGTTATTAAATGGCTAGTGTTTTTATTTGCGGCATTGTACTAAACGAAATAACAAAACAAAATAGAGAGATGATTTTCTGTA**

**Ull-2-5 FT** **CAATAAGCAAACAAAGTGAGTTATTAAATGGCTAGTGTTTTTATTTGCGGCATTGTACTAAACGAAATAACAAAACAAAATAGAGAGATGATTTTCTGTA**

**Est-1 FT**  **CAATAAGCAAACAAAGTGAGTTATTAAATGGCTAGTGTTTTTATTTGCGGCATTGTACTAAACGAAATAACAAAACAAAATAGAGAGATGATTTTCTGTA**

-6675

....|....|....|....|....|....|....|....|....|....|....|....|....|....|....|....|....|....|....|....|

**Col-0 FT**  **ATTGTAAAAATAGGAGAAACCAATGAACTGACGTTTTATTCAGTGTTCACTTTTCAGTAA-TAGGAATCAGATAAAGAATAT-TTCTTTTATTTTCAAAA**

**Ull-2-5 FT** **ATTGTAAAAATAGGAAAAACCAATGAACTGACGTTTTATTCAGTGTTCACTTTTCAGTAACTAGGAATCAGATAAAGCACATATTCTTTTATTTTCAAAA**

**Est-1 FT**  **ATTGTAAAAATAGGAGAAACCAATGAACTGACGTTTTATTCAGTGTTCACTTTTCAGTAA-TAGGAATCAGATAAAGAATAT-TTCTTTTATTTTCAAAA**

-6577

....|....|....|....|....|....|....|....|....|....|....|....|....|....|....|....|....|....|....|....|

**Col-0 FT**  **TTTTTTAAAAATATCCCAAAAAGGGTGATAAGATTTGGTGGCCATTAAAAATGCTACTTTTATGTGTGAGCTTTTTGTATTCATTCCCACGAGGCTACCC**

**Ull-2-5 FT** **TTTTTTAAAAATATCCCAAAAAGGGTGATAAGATTTGGTGGCCATTAAAAATGCTAATTTTATGTGTGAGCTTTTTGTATTCATTCCCACGAGGCTACCC**

**Est-1 FT**  **TTTTTTAAAAATATCCCAAAAAGGGTGATAAGATTTGGTGGCCATTAAAAATGCTACTTTTATGTGTGAGCTTTTTGTATTCATTCCCACGAGGCTACCC**

-6477

....|....|....|....|....|....|....|....|....|....|....|....|....|....|....|....|....|....|....|....|

**Col-0 FT**  **TCACGGCTCAAGCATCTCTCGTCATGCTTCTGACGTTCTTCATCTCCCTTTGGATATGCCATTTGCATGATATGATACATTATACATTGATAGCTTCATA**

**Ull-2-5 FT** **TCACGGCTCAAGCATCTCTCGTCATGCTTCTGACGTTCTTCATCTCCCTTTGGATATGCCATTTGCATGATATGATACATTATACATTGATAGCTTCATA**

**Est-1 FT**  **TCACGGCTCAAGCATCTCTCGTCATGCTTCTGACGTTCTTCATCTCCCTTTGGATATGCCATTTGCATGATATGATACATTATACATTGATAGCTTCATA**

-6377

....|....|....|....|....|....|....|....|....|....|....|....|....|....|....|....|....|....|....|....|

**Col-0 FT**  **CTAAATAATGCGTTTTAAAGGCTTAATAACAAATTAGTATAATGTAAAATGCTATTTGAATTTATTCTGTATATAATGTTTTTGAAAAACTTAGGAAATA**

**Ull-2-5 FT** **CTAAATAATGCGTTTTAAAGGCTTAATAACAAATTAGTATAATGTAAAATGCTATTTGAATTTATTCTGTATATAATGTTTTTGAAAAACTTAGGAAATA**

**Est-1 FT**  **CTAAATAATGCGTTTTAAAGGCTTAATAACAAATTAGTATAATGTAAAATGCTATTTGAATTTATTCTGTATATAATGTTTTTGAAAAACTTAGGAAATA**

-6277

....|....|....|....|....|....|....|....|....|....|....|....|....|....|....|....|....|....|....|....|

**Col-0 FT**  **GTGATGCATTGCAACAAATTATACTAGAAAATATCATGCTTATACCATTTATGGTGACGGCATAAAAAACTTGTGCAGTTGGAACCTTAAAAACGATATT**

**Ull-2-5 FT** **GTGATGCATTGCAACAAATTATACTAGAAAATATCATGCTTATGCCATTTATGGTGACGGCATAAAAAACTTGTGCAGTTGGAACCTTAAAAACGATATT**

**Est-1 FT**  **GTGATGCATTGCAACAAATTATACTAGAAAATATCATGCTTATACCATTTATGGTGACGGCATAAAAAACTTGTGCAGTTGGAACCTTAAAAACGATATT**

-6177

....|....|....|....|....|....|....|....|....|....|....|....|....|....|....|....|....|....|....|....|

**Col-0 FT**  **TACGTAAATTATGACACTAAATGGTGCGTTTAAACGATTAAACGTTTATATTTATTGCAATACATCACTATATTGCAACAAATTATCTAAAATATCCAAC**

**Ull-2-5 FT** **TACGTAAATTATGACACTAAATGATGCGTTTAAACGATTAAACGTTTATATTTATTGCAATACATCACTATATTGCAACAAATTATCTAAAATATCCAAC**

**Est-1 FT**  **TACGTAAATTATGACACTAAATAGTGCGTTTAAACGATTAAACGTTTATATTTATTGCAATACATCACTATATTGCAACAAATTATCTAAAATATCCAAC**

-6077

....|....|....|....|....|....|....|....|....|....|....|....|....|....|....|....|....|....|....|....|

**Col-0 FT**  **CTATGCTATTATTCATAATGGCGGCATAAAACTTGTGCAATTAGACCCTTTTAAATATTCTAAGTAATTACGTCAAAAAAAAAAAAATTACACTAGATAT**

**Ull-2-5 FT** **CTATGCTATTATTCATAATGGCGGCATAAAACTTGTGCAATTAGACCCTTTTAAATATTCTAAGTAATTACGTCAAAAAAAAATAAATTACACTAGATAT**

**Est-1 FT**  **CTATGCTATTATTCATAATGGCGGCATAAAACTTGTGCAATTAGACCCTTTTAAATATTCTAAGTAATTACGTCAAAAAAAAAAAAATTACACTAGATAT**

-5977

....|....|....|....|....|....|....|....|....|....|....|....|....|....|....|....|....|....|....|....|

**Col-0 FT**  **TTGTTTTCATTGAAAAAGATCACAAACTCAACGTCAACACAGGATGGTCTAAATTAATCAACGTGGAGATGAAAGTTAAGAGCTATTTTAAAGGCACAAA**

**Ull-2-5 FT** **TTGTTTTCATTGAAAAAGATCACAAACTCAACGTCAACACAGGATGGTCTAAATTAATCAACGTGGAGATGAAAGTTAAGAGTTATTTTAAAGGCACAAA**

**Est-1 FT**  **TTGTTTTCATTGAAAAAGATCACAAACTCAACGTCAACACAGGATGGTCTAAATTAATCAACGTGGAGATGAAAGTTAAGAGCTATTTTAAAGGCACAAA**

-5877

....|....|....|....|....|....|....|....|....|....|....|....|....|....|....|....|....|....|....|....|

**Col-0 FT**  **TTTTTATGTTAAAGACTTTTGTTCCTAACATTCATTTCCACTTCCAATATAAGATAATTCTAACATTAAAAACTTATGGTGAAAAAATATTTTTTAAAAA**

**Ull-2-5 FT** **TTTTTATGTTAAAGACTTTTGTTCCTAACATTCATTTCCACTTCCAATATAAGATAATTCTAACAATAAAAACTTATGGTGAAAAAATATTTTTTAAAAA**

**Est-1 FT**  **TTTTTATGTTAAAGACTTTTGTTCCTAACATTCATTTCCACTTCCAATATAAGATAATTCTAACATTAAAAACTTATGGTGAAAAAATATTTTTTAAAAA**

-5777

....|....|....|....|....|....|....|....|....|....|....|....|....|....|....|....|....|....|....|....|

**Col-0 FT**  **TTTTATCTGATTTGGGGTTCAAAAATATTATAAATAAAAATTCCATAATTTCTAACGAACATTTGCTGAACAAAAATCTATTACTTATTGAACCAAAAAA**

**Ull-2-5 FT** **TTTTATTTGATTTGGGGTTCAAAAATATTATAAATAAAAATTCCATAATTTCTAACGAACATTTGCTGAACAAAAATCTATTACTTATTGAACCAAAAAA**

**Est-1 FT**  **TTTTATCTGATTTGGGGTTCAAAAATATTATAAATAAAAATTCCATAATTTCTAACGAACATTTGCTGAACAAAAATCTATTACTTATTGAACCAAAAAA**

-5677

....|....|....|....|....|....|....|....|....|....|....|....|....|....|....|....|....|....|....|....|

**Col-0 FT**  **AAAATATGCTATTTTTCTCATTGAAAATCTCATTGTTTTTAACCTCTCTTCGAATTACATTCGTATGAATCACAAAAGTTTATATTTAGGAGCAGTCAAT**

**Ull-2-5 FT** **AAAA--TGCTATTTTTCTCATTGAAAATCTCATTGTTTTTAACCTCTCTTCGAATTACATTCGTATGAATCACAAAAGTTTGTATTTAGGAGCAGTCAAT**

**Est-1 FT**  **AAAATATGCTATTTTTCTCATTGAAAATCTCATTGTTTTTAACCTCTCTTCGAATTACATTCGTATGAATCACAAAAGTTTATATTTAGGAGCAGTCAAT**

-5577

....|....|....|....|....|....|....|....|....|....|....|....|....|....|....|....|....|....|....|....|

**Col-0 FT**  **AATTTATTTATTCCATTTATATTATCATCGTTAAATAAAATGTTGACAACCAACAACAAATTCAATCGGAATCAGTTCGACTGAAATTATGTTTAATTTC**

**Ull-2-5 FT** **AATTTATTTATTCCATTTATATTATCATCGTTAAATAAAATGTTGACAACCAACAACAAATTCAATCGGAATCAGTTCGACTGAAATTATGTTTAATTTC**

**Est-1 FT**  **AATTTATTTATTCCATTTATATTATCATCGTTAAATAAAATGTTGACAACCAACAACAAATTCAATCGGAATCAGTTCGACTGAAATTATGTTTAATTTC**

-5477

....|....|....|....|....|....|....|....|....|....|....|....|....|....|....|....|....|....|....|....|

**Col-0 FT**  **TTAAAGATCAAAAGTCCAAAGAAGAGATGATGAATTGAAAATAATGAGATCTTTCTTGTTGTTAAAGATAATGAGATCTTTCACATTATT**

**Ull-2-5 FT** **TTAAAGATCAAAAGTCCAAAGAAGAGATGATGAATTGAAGTTTATCAAGAATAATGAGATCTTTCTTGTTGTTAAAGATAATGAGGTCTTTCACATTATT**

**Est-1 FT**  **TTAAAGATCAAAAGTCCAAAGAAGAGATGATGAATTGAAGTTTATCAAGAATAATGAGATCTTTCTTGTTGTTAAAGATAATGGGATCTTTCACATTATT**

**I box**

-5377

....|....|....|....|....|....|....|....|....|....|....|....|....|....|....|....|....|....|....|....|

**Col-0 FT**  **GAGAGAGTATGAAGATAGATACGTTGATGATAGTGAAGTGAGACATCTTGGCCAACATTAGAAGAAGATTCCATAAAGGATTGGATGAGTGCAAAGTATC**

**Ull-2-5 FT** **GAGAGAGTATGAAGATAGATACGTTGATGATAGTGAAGTGAGACATCTTGGCCAACATTAGAAGAAGATTCCATAAAGGATTAGATGAGTGCAAAGTATC**

**Est-1 FT**  **GAGAGAGTATGAAGATAGATACGTTGATGATAGTGAAGTGAGACATCTTGGCCAACATTAGAAGAAGATTCCATAAAGGATTGGATGAGTGCAAAGTATC**

**CCAAT box**

-5277

....|....|....|....|....|....|....|....|....|....|....|....|....|....|....|....|....|....|....|....|

**Col-0 FT**  **GAGACGTCTCTACTTGGTTGCGTTTCTT-CTATTTTTATATTCGATTGTTTTACACTACTTCCTATTTTTATATT-------------------------**

**Ull-2-5 FT** **GAGACGTCTCTACTTGGTTGCGTTTCTT-CTATTTTTATATTCGATTGTTTTACACTACTTCCTATTTTTATATTCGTTTTTACATACTTCCTATTTTTA**

**Est-1 FT**  **GAGACGTCGCTACTTGGTTGCGTTTCTTTCTATTTTTATATTCGATTGTTTTACACTACTTCCTATTTTTATATTTTATATTCGTTTTTACATACTTCCT**

**REalpha**

-5207

....|....|....|....|....|....|....|....|....|....|....|....|....|....|....|....|....|....|....|....|

**Col-0 FT**  **----CGTTTTTACATACTTCCTATTTCCAAACGTTTTTTAAAATTTATTTATTAATTTCTCCTCCACATGATTTTCCTTTTGGTCTTATTCTTATTTCTT**

**Ull-2-5 FT** **TATTCGTTTTTACATACTTCCTATTTCCAACCGTTTTTTAAAATTTATTTATTAATTTCTCCTCCACATGATTTTCCTTTTGGTCTTATTCTTATTTCTT**

**Est-1 FT**  **ATTTCGTTTTTACATACTTCCTATTTCCAACCGTTTTTTAAAATTTATTTATTAATTTCTCCTCCACATGATTTTCCTTTTGGTCTTATTCTTATTTCTT**

-5107

....|....|....|....|....|....|....|....|....|....|....|....|....|....|....|....|....|....|....|....|

**Col-0 FT**  **TCGCTCCATGTCAAGAAAATATGAACTAAATAACCCAAAGACTATCATTTTATTTGTAACAATAATAAGTGGAAACATCGTAAACTGACGGTTTCGTTAA**

**Ull-2-5 FT** **TCGCTCCATGTCAAGAAAATATGAACTAAATAACCCAAAGACTATCATTTTATTTGTAACAATAATAAGTGGAAACATCGTAAACTGACGGTTTCGTTAA**

**Est-1 FT**  **TCGCTCCATGTCAAGAAAATATGAACTAAATAACCCAAAGACTATCATTTTATTTGTAACAATAATAAGTGGAAACATCGTAAACTGACGGTTTCGTTAA**

-5007

....|....|....|....|....|....|....|....|....|....|....|....|....|....|....|....|....|....|....|....|

**Col-0 FT**  **TGTTTTTGAGCTTTTATAAAAATAACATTAATTTTGTATTTATTAAATAAATATAATTATATATCTTTCCGATGACATGGTAAATTGCATGATATTATAT**

**Ull-2-5 FT** **TGCTTTTGAGCTTTTATAAAAATAACATTAATTTTGTATTTATTAAATAAATATAATTATATATCTTTCCGATGACATGGTAAATTGCATGATATTATAT**

**Est-1 FT**  **TGTTTTTGAGCTTTTATAAAAATAACATTAATTTTGTATTTATTAAATAAATATAATTATATATCTTTCCGATGACATGGTAAATTGCATGATATTATAT**

-4907

....|....|....|....|....|....|....|....|....|....|....|....|....|....|....|....|....|....|....|....|

**Col-0 FT**  **TATTTATTTAAATTTATATATACGAAAAACGTAACTTTCTTACGGTTTTCTCAGGGCAATTTTTTCCCTAGTTTTTTAAAATATAATGTTATTTTAAA--**

**Ull-2-5 FT** **TATT-ATTTAAAATTATATATACGAAAA-CGTAACTTTTTTACGGTTTTCTCAGGGCAATTTTTTCCCTAGTTTTTTAAAATACGATGTTATTTTAAAA-**

**Est-1 FT**  **TATT-ATTTAAATTTATATATACGAAAAACGTAACTTTTTTACGGTTTTCTCAGGGCAATTTTTTCCCTAGTTTTTTAAAATACGATGTTATTTTAAAAT**

-4809

....|....|....|....|....|....|....|....|....|....|....|....|....|....|....|....|....|....|....|....|

**Col-0 FT**  **GTAATCCTTTTAGAACAAGGATGCTCAATTTTTGACTAGATCCACGTAGGAAGTAGAGAAGAAAAGAAAAATTTGAGGCCCAAGTTCAGGAACTGTTGAT**

**Ull-2-5 FT** **TTAATCCTTTTAGAACAAGGATGCTCAATTTTTGACTAGATCCACGTAGGAAGTAGAGAAGAAAAGAAAAATTTGAGGCCCAAGTTCAGGAACTGTTGAT**

**Est-1 FT**  **GTAATCCTTTTAGAACAAGGATGCTCAATTTTTGACTAGATCCACGTAGGAAGTAGAGAAGAAAAGAAAAATTTGAGGCCCAAGTTCAGGAACTGTTGAT**

-4709

....|....|....|....|....|....|....|....|....|....|....|....|....|....|....|....|....|....|....|....|

**Col-0 FT**  **ATTTTTTAGCCACCAACTTCACAAGAGATATAAACACACACACACATGGG----------AACAAGACGACTGAACAAATAAGAATTCTTTT-AAGAAAT**

**Ull-2-5 FT** **ATTTTATAGCCACGAACTTCACAAGAGATATAAACACACACGCACGAGAGCCAAAAAGCGAACAAGACGACTGAACAAATAAGAATTCTTTTCAAAAAAT**

**Est-1 FT**  **ATTTTATAGCCACCAACTTCACAAGAGATATAAACACACACGCACGAGAGCCAAAAAGCGAACAAGACGACTGAACAAATAAGAATTCTTTT-AAGAAAT**

-4620

....|....|....|....|....|....|....|....|....|....|....|....|....|....|....|....|....|....|....|....|

**Col-0 FT**  **AGAGTACGACAAAAACGTGAGACGCAAAAAAAAAAAAAAAAAACTTGTAATATTCTATGATTTCTCATGAACCGAATTTATTCAGTTGCTATGATGATTT**

**Ull-2-5 FT** **AGAGTACGACAAAAACGTGAGACGCAAAAAAAAAAT----AAACTTGTAATATTCTATGATTTCTCATGAACCGAATTTATTCAGTTGCTCTGATGATTT**

**Est-1 FT**  **AGAGTACGACAAAAACGTGAGACGCAAAAAAAAAAAAAAAAAACTTGTAATATTCTATGATTTCTCATGAACCGAATTTATTCAGTTGCTATGATGATTT**

-4520

....|....|....|....|....|....|....|....|....|....|....|....|....|....|....|....|....|....|....|....|

**Col-0 FT**  **TGAGGGTCACTTTTAATAGAAAGGATTGTGGGGGTTCACTATCTTTTAGGTTCTTCTACATTTTAGTGTTCGTAACAAGCACTTATGTGTGTTAAGTCTT**

**Ull-2-5 FT** **TGAGGGTCACTTTTAATAAAAAGGATTGTGGGGGTTCACTATCTTTTAGGTCCTTCTACATGTTAGTGTTCGTAACAAGCACTTATGTGTGTTAAGTCTT**

**Est-1 FT**  **TGAGGGTCACTTTTAATAGAAAGGATTGTGGGGGTTCACTATCTTTTAGGTTCTTCTACATTTTAGTGTTCGTAACAAGCACTTATGTGTGTTAAGTCTT**

-4420

....|....|....|....|....|....|....|....|....|....|....|....|....|....|....|....|....|....|....|....|

**Col-0 FT**  **ATAAAGTTAGTTAGATAATTGTAAGTTGTTAGCTTCACATAATTAATTTTATCGATGTTACTAATACCCCACATTTTTTGCGAATCTAAGTTGCAGAAAA**

**Ull-2-5 FT** **ATAAAGTTAGTTAGATAATTGTAAGTTGTTAGCTTCACATAATTAATTTTATCGATGTTACTATTCCCCCAT-TTTTTTGCGAATTTAAGTTGCAGAAAA**

**Est-1 FT**  **ATAAAGTTAGTTAGATAATTGTAAGTTGTTAGCTTCACATAATTAATTTTATCGATGTTACTAATACCCCACATTTTTTGCGAATCTAAGTTGCAGAAAA**

-4320

....|....|....|....|....|....|....|....|....|....|....|....|....|....|....|....|....|....|....|....|

**Col-0 FT**  **TAATTGACGTAGTTTACACATTTAACTTCATAAGTAAATAAAATATGATGGAGAGATGAAATTTAATTCAAATTTGTAGCACAAGATCTTTTGGAGAACT**

**Ull-2-5 FT** **TAATTGACGTAGTTTACACATTTAACTTCAGAAGTAAATAAAATATGATGGAGAGATGAAATTTAATTCAAATTTGTAGCACAAGATTTTTTGGAGAACT**

**Est-1 FT**  **TAATTGACGTAGTTTACACATTTAACTTCATAAGTAAATAAAATATGATGGAGAGATGAAATTTAATTCAAATTTGTAGCACAAGATCTTTTGGAGAACT**

-4220

....|....|....|....|....|....|....|....|....|....|....|....|....|....|....|....|....|....|....|....|

**Col-0 FT**  **CACAACAAGTTTAGATTTAGTTTTAGGCAACGAGATTTGGGGTTAAGGTATAGGGGTTAAGCATATTAATTTATTTGGAGTTTTAACTCAAAATCAAATA**

**Ull-2-5 FT** **CACAACAAGTTTAGATTTAGTTTTAGGCAACGAGATTTGGGGTTAAGGTATAGGGGTTAAGCATATTAATTTATTTGGAGTTTTAACTCAAAATCAAATA**

**Est-1 FT**  **CACAACAAGTTTAGATTTAGTTTTAGGCAACGAGATTTGGGGTTAAGGTATAGGGGTTAAGCATATTAATTTATTTGGAGTTTTAACTCAAAATCAAATA**

-4120

....|....|....|....|....|....|....|....|....|....|....|....|....|....|....|....|....|....|....|....|

**Col-0 FT**  **GTAAAGAATGGATTGACCATAATTAAGTATTTTTCCATCT-ATCTATATGGGGCTCCACATGCCTTAAGAGATCGTGGTTTTTTAGATTTTT--------**

**Ull-2-5 FT** **GTAAAGAATGGATTGACCATAATTAAGTATTTTTCCATTTTATCTATATGGGGCTCCACATGCCTTAAGAGATAATGGTTTTTTAGATATTTTTTT----**

**Est-1 FT**  **GTAAAGAATGGATTGACCATAATTAAGTATTTTTCCATCT-ATCTATATGGGGCTCCACATGCCTTAAGAGATCGTGGTTTTTTAGATTTTTAAAAAAAA**

-4033

....|....|....|....|....|....|....|....|....|....|....|....|....|....|....|....|....|....|....|....|

**Col-0 FT**  **---------TTTTTAAAACTCATCATAATGTACTTCTAAATCTCGACAAAATGAAATCAAGCTTTTGTT--GGACATTCAGTTCCGGACAATCGGACTGC**

**Ull-2-5 FT** **---------TTTTTGAAACTCATCATAATGTACTTTTAAATCCCGACAAAATGAAATCAAGCTTTTGTTCAGGACATTCAGTTCCGGACAATCGGACTGT**

**Est-1 FT**  **AAAAAAAAATTTTTAAAACTCATCATAATGTACTTCTAAATCTCGACAAAATGAAATCAAGCTTTTGTTCAGGACATTCAGTTCCGGACAATCGGACTGC**

-3940

....|....|....|....|....|....|....|....|....|....|....|....|....|....|....|....|....|....|....|....|

**Col-0 FT**  **ATTCAAATAACTTGTTTTCGGACTTGGATATGATGTTAAGTATCATATATTCATATGCGTCCAACTAAATATCAATTGACAAAAATTGGATTGACACATA**

**Ull-2-5 FT** **ATTCAAATAACTTGTTTTCGGACTTGGATATGATGTTAAGTATCA-------------------------------------------------------**

**Est-1 FT**  **ATTCAAATAACTTGTTTTCGGACTTGGATATGATGTTAAGTATCATATATTCATATGCGTCCAACTAAATATCAATTGACAAAAATTGGATTGACACATA**

-3840

....|....|....|....|....|....|....|....|....|....|....|....|....|....|....|....|....|....|....|....|

**Col-0 FT**  **TCTCTTATATACTAGTGTTTCTTTTTAAAACTTTCAATGTGGGATCTTATTAAAAGAAATCCTCTTCTCAAAACTTTATAAACTTTCATTAGACTAATTA**

**Ull-2-5 FT** **----------------------------------------------------------------------------------------------------**

**Est-1 FT**  **TCTCTTATATACTAGTGTTTCTTTTTAAAACTTTCAATGTGGGATCTTATTAAAAGAAATCCTCTTCTCAAAACTTTATAAACTTTCATTAGACTAATTA**

-3740

....|....|....|....|....|....|....|....|....|....|....|....|....|....|....|....|....|....|....|....|

**Col-0 FT**  **TAAGCTAAACTATCTAAAATCTCTTACATATTATTTGAGAAGTCGCAATTTTTTTTGTCATCTCACTTTATTGAGTAAAGAATAATATCAAACATAAAAC**

**Ull-2-5 FT** **----------------------------------------------------------------------------------------------------**

**Est-1 FT**  **TAAGCTAAACTATCTAAAATCTGTTA--TATTATTTGAGAAGTCGCAATTTTTTTTGTCATCTCACTTTATTGAGTAAAGAATAATATCAAACATAAAAC**

-3640

....|....|....|....|....|....|....|....|....|....|....|....|....|....|....|....|....|....|....|....|

**Col-0 FT**  **AAGTTAATCTAAGACTTGCAGATAATAAACCAACATGGGAAGATAATTGAAGGATTATCTTCTAGCTTAGAGAACATGTGTCGCAATTAATTTGATGACA**

**Ull-2-5 FT** **----------------------------------------------------------------------------------------------------**

**Est-1 FT**  **AAGTTAATCTAAGACTTGCAGATAATAAACCAACATGGGAAGATAATTGAAGGATTATCTTCTAGCTTAGAGAACATGTGTCGCAATTAATTTGATGACA**

-3540

....|....|....|....|....|....|....|....|....|....|....|....|....|....|....|....|....|....|....|....|

**Col-0 FT**  **AAGGGCACTCATGAGGATATGATTTAGAAATGTCATCGAATACTGACGATCGATTTTTTCGAGTCCGATGAATTACAATTCACAGAGGTAAAGAAATCTG**

**Ull-2-5 FT** **----------------------------------------------------------------------------------------------------**

**Est-1 FT**  **AAGGGCACTCATGAGGATATGATTTAGAAATGTCATCGAATACTGACGATCGATTTTTTCGAGTCCGATGAATTACAATTCACAGAGGTAAAGAAATCTG**

-3440

....|....|....|....|....|....|....|....|....|....|....|....|....|....|....|....|....|....|....|....|

**Col-0 FT**  **CTTTTGAATCACCGATTCTTCTAACTTCTATTCAGATGATAGGTTAAATGAAGAAGAAGAAAAAAAGAGAAGAAAATCTATCAAGATCCCTTATGCTAGC**

**Ull-2-5 FT** **-----------------------------------------------------------------------------------AGATCCCTTATGCTAGC**

**Est-1 FT**  **CTTTTGAATCACCGATTCTTCTAACTTCTATTCAGATGATAGGTTAAATGAAGAAGAAGAAAAAA-GAGAAGAAAATCTATCAAGATCCCTTATGCTAGC**

-3340

....|....|....|....|....|....|....|....|....|....|....|....|....|....|....|....|....|....|....|....|

**Col-0 FT**  **CCAAAATCACATCAAGTTTTTCACATAATGAGATTTAGCAAAAACTTGTAAACGTAGGTTTGCCGGATCTCTTGTTCCTCATCCACTTGCCAA--TCTTC**

**Ull-2-5 FT** **C-AAAATCACATCAAGTTTTTCACATAATGAAATTTAGCAAAAACTTGTAAACGTAGGTTTGCCGGATCTCTTATTCCTCATCCCCTTGCCAAGATCTTC**

**Est-1 FT**  **CCAAAATCACATCAAGTTTTTCACATAATGAGATTTAGCAAAAACTTGTAAACGTAGGTTTGCCGGATCTCTTGTTCCTCATCCACTTGCCAA--TCTTC**

-3242

....|....|....|....|....|....|....|....|....|....|....|....|....|....|....|....|....|....|....|....|

**Col-0 FT**  **GTAATCCAAAAGGTAAATATCTATGTTTAATTATCTTCTAGATTTTAAGGCACAACGGATTGATCGTTCTATATTGTATGCACGATTGAAGAAGTTGTTT**

**Ull-2-5 FT** **GTAATCCAAAAGGTAAATATGTATGTTTAATTATCTTCC-------------------------------------------------------------**

**Est-1 FT**  **GTAATCCAAAAGGTAAATATCTATGTTTAATTATCTTCTACATTTTAAGGCACAACGGATTGATCGTTCTATATTGTATGCACGATTGAAGAAGTTGTTT**

-3142

....|....|....|....|....|....|....|....|....|....|....|....|....|....|....|....|....|....|....|....|

**Col-0 FT**  **AAGAAGCACAAACATTTGACTATCGGCTTTAACACCTTTTTGTCACTTGGAGATAAGATATTTCTTCATGGTGACGCAGAAGCCTCTACTAGCTCCACCG**

**Ull-2-5 FT** **----------------------------------------------------------------------------------------------------**

**Est-1 FT**  **AAGAAGCACAAACATTTGACTATCGGCTTTAACACCTTTTTGTCACTTGGAGATAAGATATTTCTTCATGGTGACGCAGAAGCCTCTACTAGCTCCACCG**

-3042

....|....|....|....|....|....|....|....|....|....|....|....|....|....|....|....|....|....|....|....|

**Col-0 FT**  **CAAAAGACTATTGAAATTTCTTATCATGGTCATTACACCAATGATACTATCAAAATCTCTAAACTAATCTCTATGTTCAGTTTTTGATGGTTTATTATCA**

**Ull-2-5 FT** **----------------------------------------------------------------------------------------------------**

**Est-1 FT**  **CAAAAGACTATTGAAATTTCTTATCATGGTCATTACACCAATGATACTATCAAAATCTCTAAACTAATCTCTATGTTCAGTTTTTGATGGTTTATTATCA**

-2942

....|....|....|....|....|....|....|....|....|....|....|....|....|....|....|....|....|....|....|....|

**Col-0 FT**  **AGTATGTGTCTTTGTAATTATCTTCTCTTCTAAGATTGAATCATCACAATGTTTTTGAATTATTTGATTTTCATTGAATATTTCAACCAACCAAAATGTT**

**Ull-2-5 FT** **--------------------------CTTCTATAATTGAATCATCTCAATGTTTTTGAATTATTTGATTTTCATTGAATATTTCAACCAACCAAAATGTT**

**Est-1 FT**  **AGTATGTGTCTTTGTAATTATCTTCTCTTCTAAGATTGAATCATCACAATGTTTTTGAATTATTTGATTTTCATTGAATATTTCAACCAACCAAAATGTT**

-2842

....|....|....|....|....|....|....|....|....|....|....|....|....|....|....|....|....|....|....|....|

**Col-0 FT**  **TTCGTTCTAGAAATAAAGTCCTAAAGTGACGTACATTCGTTTTCTAATGAAAATGCTATTTCATAAGTTATTCCTTTCATTTTTTTTGGGTCGTCAAAAC**

**Ull-2-5 FT** **TTCGTTCTAAAAATAAAGTCCTAAAGTGACGTACATTCGTTTTCTAATGAAAATGCTATTTCATAAGTTATTCTTTTCAATTTTTTT--TTGGCCAAAAC**

**Est-1 FT**  **TTCGTTCTAGAAATAAAGTCCTAAAGTGACGTACATTCGTTTTCTAATGAAAATGCTATTTCATAAGTTATTCCTTTCATTTTTTTTGGGTCGTCAAAAC**

-2742

....|....|....|....|....|....|....|....|....|....|....|....|....|....|....|....|....|....|....|....|

**Col-0 FT**  **ATAAGTTAATATTCTTTTTTTATTTGACTAAAACTACTTTTCCAAAATTTTCACCGGGGAAACCTTCTCAAAGCTTTATGAGCTTAAACATATGCAAAGT**

**Ull-2-5 FT** **ATAAGTTAATATTCTTTTTTTATTTGACTAAAACTACTTTTCCAAAATTTTCACCGGGGAAACCTTCTCAAAGCTTTATGAGCTTAAACATATGCAAAGT**

**Est-1 FT**  **ATAAGTTAATATTCTTTTTTTATTTGACTAAAACTACTTTTCCAAAATTTTCACCGGGGAAACCTTCTCAAAGCTTTATGAGCTTAAACATATGCAAAGT**

-2642

....|....|....|....|....|....|....|....|....|....|....|....|....|....|....|....|....|....|....|....|

**Col-0 FT**  **GTCTCATTAACTTAAATAAATATAGAGTACAATTAATTTGTTCACAACGTTTTTAATAGGAAACTTAGAATATGTTCTGTGATGCACTATATGTTGCTTC**

**Ull-2-5 FT** **GTCTCATTAACTTAAATAAATATAGAGTACAATTAATTTGTTCACAACGTTTTTAATAGGAAACTTAGAATATGTTCTGTGATGCACTATGTGTTGCTTC**

**Est-1 FT**  **GTCTCATTAACTTAAATAAATATAGAGTACAATTAATTTGTTCACAACGTTTTTAATAGGAAACTTAGAATATGTTCTGTGATGCACTATATGTTGCTTC**

-2542

....|....|....|....|....|....|....|....|....|....|....|....|....|....|....|....|....|....|....|....|

**Col-0 FT**  **GTAAGGAAATTGCATGCGAAAATCTAGTGGAAGAAATTTAGTTAAACGGCAAAAATCTTCAATTAATCGTTTCCTTTATCTACATTACCAAAAGGTTGCG**

**Ull-2-5 FT** **GTAAGGAAATTGCATGCGAAAATCTAGTGGAAGAAATTTAGTTAAACGGCAAAAATCTTCAATTAATCGTTTCCTTTATCTACATTACCAAAAGGTTGCG**

**Est-1 FT**  **GTAAGGAAATTGCATGCGAAAATCTAGTGGAAGAAATTTAGTTAAACGGCAAAAATCTTCAATTAATCGTTTCCTTTATCTACATTACCAAAAGGTTGCG**

-2442

....|....|....|....|....|....|....|....|....|....|....|....|....|....|....|....|....|....|....|....|

**Col-0 FT**  **AACATTATGCGACATATGGTGGTTAGAACTTAATA------------CACTAATGTTTTTCAAAGATGCTATTTGTGGAGTTGATAAAGACAAAACATAT**

**Ull-2-5 FT** **AACATTATGCGACATATGGTGGTTAGAACTTAATATCTATCAAAATACACTAATGTTTTTCAAAGATGCTATTTGTGGAGTTGATAAAGACAAAACATAT**

**Est-1 FT**  **AACATTATGCGACATATGGTGGTTAGAACTTAATA------------CACTAATGTTTTTCAAAGATGCTATTTGTGGAGTTGATAAAGACAAAACATAT**

-2354

....|....|....|....|....|....|....|....|....|....|....|....|....|....|....|....|....|....|....|....|

**Col-0 FT**  **TTGAAGAAAATAATTTTAAACATGTAGTGTTTAGGAACATCACGGCTTCATAGATATAGAGTCAACTTAATATGTTACAATGATATTGAAATATGTTCTC**

**Ull-2-5 FT** **TTGAAGAAAATACTATTGAACATGTAGTGTTTTGGAACATCACGGCTTCATAGATATAGATTCAACTTAATATGTTACAATGATATTGAAATATGTTCTC**

**Est-1 FT**  **TTGAAGAAAATAATTTTAAACATGTAGTGTTTAGGAACATCACGGCTTCATAGATATAGAGTCAACTTAATATGTTACAATGATATTGAAATATGTTCTC**

-2254

....|....|....|....|....|....|....|....|....|....|....|....|....|....|....|....|....|....|....|....|

**Col-0 FT**  **TAAACTCTAAAAGGTTCTACCTGATTTTCTTAAACAATGCATCTTCGAAAGATAAAGAAAATATATTTTTCTATTTTCTCTTTACATGTCAATGCTTACT**

**Ull-2-5 FT** **TAAACTCTAAA-GGTTCTATCTGATTTTCTTAAACAATGCATCTTCGAAAGATAAAGAAAATATATTTTTCTATTTTCATTTTACATGTCAATGCTTACT**

**Est-1 FT**  **TAAACTCTAAAAGGTTCTACCTGATTTTCTTAAACAATGCATCTTCGAAAGATAAAGAAAATATATTTTTCTATTTTCTCTTTACATGTCAATGCTTACT**

-2154

....|....|....|....|....|....|....|....|....|....|....|....|....|....|....|....|....|....|....|....|

**Col-0 FT**  **ATATCATCTTACGATATACTATAAACGTTTTATTCATAAGTAAAAGACAAGTGGCAGATACGTTAAATTTTATAATAGAAATAAATTAGTTAAATCTTAA**

**Ull-2-5 FT** **ATATTATCTTACGATATACTATAAACGTTTTATTCATAAGTAAAAGACAAGTGGCAGATACGTTAAATTTTATAATAGAAATAAATTAGTTAAATCTTAA**

**Est-1 FT**  **ATATCATCTTACGATATACTATAAACGTTTTATTCATAAGTAAAAGACAAGTGGCAGATACGTTAAATTTTATAATAGAAATAAATTAGTTAAATCTTAA**

-2054

....|....|....|....|....|....|....|....|....|....|....|....|....|....|....|....|....|....|....|....|

**Col-0 FT**  **AAAGTTGCTGAAAATAGAAAGCAAACTACTTATCTTAAAGCAAACGTAAGTCTCTTTAAGAAGAAATACAAAATGAATATATATCGGATTAAATCAAAAA**

**Ull-2-5 FT** **AAAGTTGCTGAAAATAGAAAGCAAACTACTTATCTTAAAGCAAACGTAAGTCTCTTTAAGAAGAAATACAAAATGAATGTATATAGGATTAAATCAAAAA**

**Est-1 FT**  **AAAGTTGCTGAAAATAGAAAGCAAACTACTTATCTTAAAGCAAACGTAAGTCTCTTTAAGAAGAAATACAAAATGAATATATATCGGATTAAATCAAAAA**

-1954

....|....|....|....|....|....|....|....|....|....|....|....|....|....|....|....|....|....|....|....|

**Col-0 FT**  **ACAAAAAGATACACTTTTGTTTTTCTGAACAAATGAAAAATGATATACACAAGTGGCGGACAATCCATCTATCTCATTTTAGCGAACAATTAATCTTGTC**

**Ull-2-5 FT** **ACAAAAAGATACACTTTTGTTTTTCTGAACAAATGAAAAATGATATACACAAGTGGCGGACAATCCATCTATCTCATTTTAGCGAACAATTAATCTTGTC**

**Est-1 FT**  **ACAAAAAGATACACTTTTGTTTTTCTGAACAAATGAAAAATGATATACACAAGTGGCGGACAATCCATCTATCTCATTTTAGCGAACAATTAATCTTGTC**

-1854

....|....|....|....|....|....|....|....|....|....|....|....|....|....|....|....|....|....|....|....|

**Col-0 FT**  **TGCGACTGCGACCTATTTTTTTCTACATCACATCTTTTGTTTCGTATGAACACTAACATGATTGAATGACAAAATTTACTAACAATTCTTATAGTACTGA**

**Ull-2-5 FT** **TGCGACTGCGACCTATTTTATTCTACATCACATCTTTTGTTTCGTATGAACACTAACATGATTGAATGACAAAATTTACTAACAATTCTTATAGTACTGA**

**Est-1 FT**  **TGCGACTGCGACCTATTTTTTTCTACATCACATCTTTTGTTTCGTATGAACACTAACATGATTGAATGACAAAATTTACTAACAATTCTTATAGTACTGA**

-1754

....|....|....|....|....|....|....|....|....|....|....|....|....|....|....|....|....|....|....|....|

**Col-0 FT**  **GCTCTCCTGTCCAATATTTAAAAATGCATACATTTTTTTGTGTGTAACTAAAATTAGTAGAAAATAAATGCTAGTCGTTAAAAAGTGCATATAGCAGATT**

**Ull-2-5 FT** **GCTCTCCTGTCCAATATTTAAAAATGCATACATTTTTTTATGTGTAACTAAAATTAGTAGAAAATAAATGCTAGTCGTTAAAAAGTGCATATAGCAGATT**

**Est-1 FT**  **GCTCTCCTGTCCAATATTTAAAAATGCATACATTTTTTTGTGTGTAACTAAAATTAGTAGAAAATAAATGCTAGTCGTTAAAAAGTGCATATAGCAGATT**

-1654

....|....|....|....|....|....|....|....|....|....|....|....|....|....|....|....|....|....|....|....|

**Col-0 FT**  **TCGAGTTTTTTTTAATTGCGATTTCGATTATCAATGGTCGATCGTAGACGTCTTTTTTAGTAAACAAATCTGAAATTTCTATGAAGGTTACCAAATATTT**

**Ull-2-5 FT** **TCGAGTTTTTTTTAATTGCGATTTCGATTATCAATGGTCGATCGTAGACGTCTATTTTAGTAAAAAAATCTGAAAATTCTATGAAGGTTACCAATTATTT**

**Est-1 FT**  **TTGAGTTTTTTTTAATTGCGATTTCGATTATCAATGGTTGATCGTAGACGTCTTTTTTAGTAAACAAATCTGAAATTTCTATGAAGGTTACCAATTATTT**

-1554

....|....|....|....|....|....|....|....|....|....|....|....|....|....|....|....|....|....|....|....|

**Col-0 FT**  **TGGATTTCAACCTAAGATGAAGTTAACCTATGGACGTGTAGAACAGTGGCTGAAGTCTGAAATGATAAGATAAAGTTCAATATGAATACATTACTGAGAT**

**Ull-2-5 FT** **TGGATTTCAACCTAAGATGAAGTTAA---ATGGACGTGTAGAACAGTGGCTGAAGTCTGAAATGATAAGATAAAGTTCAATATAAATACATCACTGAGAT**

**Est-1 FT**  **TGGATTTCAACCTAAGATGAAGTTAACCTATGGACGTGTAGAACAGTGGCTGAAGTCTGAAATGATAAGATAAAGTTCAATATAAATACATCACTGAGAT**

-1454

....|....|....|....|....|....|....|....|....|....|....|....|....|....|....|....|....|....|....|....|

**Col-0 FT**  **GTTACAAAAAA----CTACATCACTGAATAATTCCTTTATTTTCCAGTTTGGACAGTAGAACCTATCGCTAATTATTTAATTACCAATTAAAA-GAAAAA**

**Ull-2-5 FT** **GTCACAAAAAAAAAACTACATCACTGAATAATTCCTTTATTTTCCAGTTTGGACAGTAGAACCTATCGTTAAATATTTAATTACCAATTAAAAAGAAAAA**

**Est-1 FT**  **GTCACAAAAAAAAAACTACATCACTGAATAATTCCTTTCTTTTCCAGTTTGGACAGTAGAACCTATCGTTAAATATTTAATTACCAATTAAAAAGAAAAA**

-1359

....|....|....|....|....|....|....|....|....|....|....|....|....|....|....|....|....|....|....|....|

**Col-0 FT**  **GAAAGAATAATTCCTACAGTTGTTAGGCTATGGTTATAAGTTTCATCTTTGAACTTAAGAAATGCTCTAATTAGTGGTAATGGTAGTTACATCTATATAT**

**Ull-2-5 FT** **GAAAGAATAATTCCTACAGTTGTTAGGCTATGGTTATAAGTTTCATCTTTGAACTTAAGAAATGCTCTAATTAGTGGTAATGGTAGTTACATCTATATAT**

**Est-1 FT**  **GAAAGAATAATTCCTACAGTTGTTAGGCTATGGTTATAAGTTTCATCTTTGAACTTAAGAAATGCTCTAATTAGTGGTAATGGTAGTTACATCTATATAT**

-1259

....|....|....|....|....|....|....|....|....|....|....|....|....|....|....|....|....|....|....|....|

**Col-0 FT**  **GTTGATGCAATGTCAAAAAGAAAATCTCTCAAATTAGCAAATCCATACCTATTTATAGACGCATAAGTATATATACCAATTATCCTGGTCGTGCAAATGG**

**Ull-2-5 FT** **GTTGATGCAATGTCAAAAAGAAAATCTCTCAAATTAGCAAATCCATACCTATTTATAGACGCATAAGTATATATACCAATTATCCTGGTCGTGCAAATGG**

**Est-1 FT**  **GTTGATGCAATGTCAAAAAGAAAATCTCTCAAATTAGCAAATCCATACCTATTTATAGACGCATAAGTATATATACCAATTATCCTGGTCGTGCAAATGG**

-1159

....|....|....|....|....|....|....|....|....|....|....|....|....|....|....|....|....|....|....|....|

**Col-0 FT**  **ATGGTTAGTATTTTTACAACCAAACTAATGTCTAATTTTAAAAAGTAATCATTATTTTGCTTTAGATTTTTAATAGTTAATATTATATGTATAGATAGAT**

**Ull-2-5 FT** **ATGGTTAGTATTTTTACAACCAAACTAATGTCTAATTTTAAAAAGTAATCATTATTTTGCTTTAGATTTTTAATAGTTAATATTATATGTATAGATAGAT**

**Est-1 FT**  **ATGGTTAGTATTTTTACAACCAAACTAATGTCTAATTTTAAAAAGTAATCATTATTTTGCTTTAGATTTTTAATAGTTAATATTATATGTATAGATAGAT**

-1059

....|....|....|....|....|....|....|....|....|....|....|....|....|....|....|....|....|....|....|....|

**Col-0 FT**  **TACCGTATGATATAATTGTTTCAAGACACTAGATACTATCTCAATTATAAAACTCACTTTTCCATAATATGGCCGCTTGTTTATAAAAAAAGAAGAGAAA**

**Ull-2-5 FT** **TACCGTATGATATAATTGTTTCAAGACACTAGATACTATCTCAATTATAAAACTCACTTTTCCATAATATGGCCGCTTGTTTATAAAAAAAGAAGAGAAA**

**Est-1 FT**  **TACCGTATGATATAATTGTTTCAAGACACTAGATACTATCTCAATTATAAAACTCACTTTTCCATAATATGGCCGCTTGTTTATAAAAAAAGAAGAGAAA**

-959

....|....|....|....|....|....|....|....|....|....|....|....|....|....|....|....|....|....|....|....|

**Col-0 FT**  **TAAAACAATTGATTTGGTTTATATTATTTAATTGCAGATATCTTGTACTTAATTCATTTTGAGATAATTTTGCGTATTTGAGTTCGGACATTGGTAGGTA**

**Ull-2-5 FT** **TAAAACAATTGATTTGGTTTATATTATTTAATTGCAGATATCTTGTACTTAATTCATTTTGAGATAATTTTGCGTATTTGAGTTCGGACATTGGTAGGTA**

**Est-1 FT**  **TAAAACAATTGATTTGGTTTATATTATTTAATTGCAGATATCTTGTACTTAATTCATTTTGAGATAATTTTGCGTATTTGAGTTCGGACATTGGTAGGTA**

-859

....|....|....|....|....|....|....|....|....|....|....|....|....|....|....|....|....|....|....|....|

**Col-0 FT**  **TGGACGATGAAAATAACTGCCTTCATTCTACATGTTTGAGATTTGTTTGTCGACCATATAACACAAGCGGCTAGAAAAATAGGTGACTATTCTCAAATGT**

**Ull-2-5 FT** **TGGACGATGAAAATAACTGCCTTCATTCTACATGTTTGAGATTTGTTTGTCGGCCATATAACACAAACGGCTAGAAAAATAGGTGACTATTCTCAAATGT**

**Est-1 FT**  **TGGACGATGAAAATAACTGCCTTCATTCTACATGTTTGAGATTTGTTTGTCGGCCATATAACACAAACGGCTAGAAAAATAGGTGACTATTCTCAAATGT**

-759

....|....|....|....|....|....|....|....|....|....|....|....|....|....|....|....|....|....|....|....|

**Col-0 FT**  **CCTGGTTCTATCTAACCTGAAGGATCCCTTGTTCTACCTGGTATAACTACAAGAAAAGGCTGTTTTATAAAATATATCATTTCTTCTATGATTTCTATCT**

**Ull-2-5 FT** **CCTGGTTCTATCTAACCTGAAGGATCCCTTGTTCTACCTGGTATAACTACAAGAAAAGGCTGTTTTATAAAATATATCATTTCTTCTATGATTTCTATCT**

**Est-1 FT**  **CCTGGTTCTATCTAACCTGAAGGATCCCTTGTTCTACCTGGTATAACTACAAGAAAAGGCTGTTTTATAAAATATATCATTTCTTCTATGATTTCTATCT**

-659

....|....|....|....|....|....|....|....|....|....|....|....|....|....|....|....|....|....|....|....|

**Col-0 FT**  **CAACTTTTTTTTTAGTTTTTTTCTTTTAGAACGTTTTCGCTTTCGAATTTTTTAAGAATTTTTTTTTTCTTTATATCTCTAAATAATAGTTTTAAAAACA**

**Ull-2-5 FT** **CAACTTTTTTTTTTAGTTTTTTTTTTAGAACGTTTTCGCTTTCGAATTTTTTAAGAATTTTTTTTTCTTTATATCTCTAAATAATAGTTTTAAAAACATA**

**Est-1 FT**  **CAACTTTTTTTTTTAGTTTTTT------------------------------------------------------------------------------**

* The position of nucleotide is marked upstream of *FT* start codon ATG. The conserved motifs are indicated with red underlines.
